# Genetically encoded calcium indicators for multi-color neural activity imaging and combination with optogenetics

**DOI:** 10.3389/fnmol.2013.00002

**Published:** 2013-03-04

**Authors:** Jasper Akerboom, Nicole Carreras Calderón, Lin Tian, Sebastian Wabnig, Matthias Prigge, Johan Tolö, Andrew Gordus, Michael B. Orger, Kristen E. Severi, John J. Macklin, Ronak Patel, Stefan R. Pulver, Trevor J. Wardill, Elisabeth Fischer, Christina Schüler, Tsai-Wen Chen, Karen S. Sarkisyan, Jonathan S. Marvin, Cornelia I. Bargmann, Douglas S. Kim, Sebastian Kügler, Leon Lagnado, Peter Hegemann, Alexander Gottschalk, Eric R. Schreiter, Loren L. Looger

**Affiliations:** ^1^Janelia Farm Research Campus, Howard Hughes Medical InstituteAshburn, VA, USA; ^2^Medical Research Council Laboratory of Molecular BiologyCambridge, UK; ^3^Department of Chemistry, University of Puerto Rico - Río PiedrasSan Juan, PR, USA; ^4^Institute of Biochemistry and Buchmann Institute for Molecular Life Sciences, Johann Wolfgang Goethe-University FrankfurtFrankfurt, Germany; ^5^Experimentelle Biophysik, Humboldt Universität zu BerlinBerlin, Germany; ^6^Department of Neurology, University Medicine GöttingenGöttingen, Germany; ^7^Laboratory of Neural Circuits and Behavior, Howard Hughes Medical Institute, The Rockefeller UniversityNew York, NY, USA; ^8^Department of Molecular and Cellular Biology, Center for Brain Science, Harvard UniversityCambridge, MA, USA; ^9^Champalimaud Neuroscience Programme, Champalimaud Centre for the UnknownLisboa, Portugal

**Keywords:** calcium imaging, genetically encoded calcium indicator, multi-color imaging, protein engineering, optogenetics

## Abstract

Genetically encoded calcium indicators (GECIs) are powerful tools for systems neuroscience. Here we describe red, single-wavelength GECIs, “RCaMPs,” engineered from circular permutation of the thermostable red fluorescent protein mRuby. High-resolution crystal structures of mRuby, the red sensor RCaMP, and the recently published red GECI R-GECO1 give insight into the chromophore environments of the Ca^2+^-bound state of the sensors and the engineered protein domain interfaces of the different indicators. We characterized the biophysical properties and performance of RCaMP sensors *in vitro* and *in vivo* in *Caenorhabditis elegans*, *Drosophila* larvae, and larval zebrafish. Further, we demonstrate 2-color calcium imaging both within the same cell (registering mitochondrial and somatic [Ca^2+^]) and between two populations of cells: neurons and astrocytes. Finally, we perform integrated optogenetics experiments, wherein neural activation *via* channelrhodopsin-2 (ChR2) or a red-shifted variant, and activity imaging *via* RCaMP or GCaMP, are conducted simultaneously, with the ChR2/RCaMP pair providing independently addressable spectral channels. Using this paradigm, we measure calcium responses of naturalistic and ChR2-evoked muscle contractions *in vivo* in crawling *C. elegans*. We systematically compare the RCaMP sensors to R-GECO1, in terms of action potential-evoked fluorescence increases in neurons, photobleaching, and photoswitching. R-GECO1 displays higher Ca^2+^ affinity and larger dynamic range than RCaMP, but exhibits significant photoactivation with blue and green light, suggesting that integrated channelrhodopsin-based optogenetics using R-GECO1 may be subject to artifact. Finally, we create and test blue, cyan, and yellow variants engineered from GCaMP by rational design. This engineered set of chromatic variants facilitates new experiments in functional imaging and optogenetics.

## Introduction

Together, recent advances in modern microscopy and improved genetically encoded calcium indicators (GECIs) have revolutionized systems neuroscience by allowing chronic simultaneous optical recording from genetically targeted neuronal populations *in vivo*. Calcium is a ubiquitous second messenger, playing an essential role in all aspects of physiology, specifically in neurons and other excitable cells (Burgoyne, 2007). Calcium ions (Ca^2+^) are transported into neurons both by action potential (AP) firing and synaptic input (Jaffe et al., 1992; Denk et al., 1996). Spike number, timing, frequency, as well as levels of synaptic input, can all be quantified by measuring changes in intracellular free [Ca^2+^] (Yasuda et al., 2004). GECIs are prominent tools to monitor [Ca^2+^] in defined cells and intra-cellular compartments (Mank and Griesbeck, 2008; Mao et al., 2008; Dreosti et al., 2009; Hou et al., 2009; Rothermel et al., 2009; Shigetomi et al., 2010). The most optimized GECIs are single-wavelength green indicators based on the original GCaMP sensor (Nakai et al., 2001). Improvements have been facilitated both by crystal structure determination in the Ca^2+^-free and Ca^2+^-bound states (Wang et al., 2008; Akerboom et al., 2009), and high-throughput screening in bacterial colonies (Ibraheem et al., 2011; Zhao et al., 2011a; Akerboom et al., 2012b) and lysates (Tian et al., 2009). A number of engineered variants of GCaMP have been published (Ohkura et al., 2005; Tallini et al., 2006; Tian et al., 2009; Muto et al., 2011); of these the GCaMP5 indicators (Akerboom et al., 2012a) show the best performance in detecting APs in neurons. GCaMP sensors have been deployed in a number of model organisms, and facilitate experiments such as imaging Ca^2+^ transients during development in transgenic mice (Zariwala et al., 2012), *in vivo* imaging of layer 5 cortex (Mittmann et al., 2011), chronic measurements of circuit dynamics accompanying motor task learning (Huber et al., 2012), and monitoring whisker sensory-motor integration in cortical synaptic terminals (Petreanu et al., 2012).

Sufficient structure/function relationships are known for GCaMP (Wang et al., 2008; Akerboom et al., 2009) and its constituent molecules, calmodulin (CaM) (Chou et al., 2001; Faas et al., 2011; Stigler and Rief, 2012) and green fluorescent protein (GFP) (Ormo et al., 1996; Tsien, 1998), to allow specific, semi-rational manipulation of critical sensor parameters including: Ca^2+^ affinity, on- and off-kinetics, protein stability, expression/degradation profiles, and baseline and activated fluorescence levels (Akerboom et al., 2009, 2012b; Tian et al., 2009). One characteristic that until recently has largely been unexplored (Zhao et al., 2011a) is sensor color.

Modulation of the color of GFP and related proteins is well established. Direct mutation of the side-chains comprising the GFP chromophore can tune excitation/emission; specifically, the mutations Phe64Leu, Thr65Ser, and Tyr66His produce a blue fluorescent protein (BFP) (Heim et al., 1994), and Tyr66Trp produces a cyan variant (CFP) (Heim and Tsien, 1996). The Thr203Tyr mutation produces a pi-stacking interaction with the GFP chromophore that red-shifts fluorescence, creating a yellow fluorescent protein (YFP) (Ormo et al., 1996). A number of improved variants of the original BFP, CFP, and YFP have been published, often by mutating positions near the chromophore to improve folding, maturation, brightness, and photostability (Griesbeck et al., 2001; Nagai et al., 2002; Rizzo et al., 2004; Nguyen and Daugherty, 2005; Ai et al., 2006, 2007; Kremers et al., 2006, 2007; Mena et al., 2006; Goedhart et al., 2010). A red variant of GFP has also been published (Mishin et al., 2008), but it is quite dim.

Mutations producing chromatic variants of FPs can easily be grafted onto sensors derived from them. In spite of this, until recently (Zhao et al., 2011a) the only available color of single-wavelength GECI was green [camgaroo (Yu et al., 2003), pericam (Nagai et al., 2001), and the Case sensors (Leder et al., 2010) were engineered from EYFP but have GFP-like green fluorescence]. For analytes other than Ca^2+^, sensors with several different colors have been constructed: Hyper, a yellow peroxide sensor (Belousov et al., 2006); blue, cyan, green, and yellow sensors for maltose (Marvin et al., 2011); a yellow sensor of molecular strain (Ichimura et al., 2012); Frex, a yellow sensor of NADH (Zhao et al., 2011b); PermELI, a yellow estrogen sensor (Picazo et al., 2011); and a yellow indicator of ATP:ADP ratio (Berg et al., 2009).

Extending the color palette greatly increases the potential of GECIs: multi-color imaging of different cell types and organelles could reveal inter- and intra-cell signaling events; red-shifted indicators would reduce tissue scattering, phototoxicity, and background fluorescence, facilitating deep imaging; non-green sensors could be used in animals already expressing a green FP; and perhaps most importantly, color-shifted indicators could seamlessly integrate into optogenetics experiments. Optogenetic manipulation of cells *via* light-modulated ion proteins, such as the microbial opsins (Yizhar et al., 2011a) or photoactivated cyclases (Stierl et al., 2011), combined with functional imaging of genetically encoded sensors, could elucidate the input/output interactions both within single cells and between neurons. However, the GCaMP excitation spectrum overlaps the action spectra of commonly used activators and silencers, such as channelrhodopsin-2 (ChR2) (Nagel et al., 2003, 2005; Boyden et al., 2005), archaerhodopsin-3 (Arch) (Chow et al., 2010), and halorhodopsin (HR) (Zhang et al., 2007). Currently, imaging GCaMP fluorescence without overly activating ChR2 is only possible with dim excitation light, resulting in weak fluorescent signals and low signal-to-noise ratio (SNR) (Guo et al., 2009). Using a red-shifted GECI in concert with the blue-activated ChR2 could allow activating and imaging lasers to be used at full intensity.

We first sought to create a family of chromatic variants of GCaMP by structure-guided design and random screening, and compare those to the color variants recently published (Zhao et al., 2011a). Initially, we made mutations in and around the chromophore in GCaMP3, by grafting mutations of GFP that produce blue (Heim et al., 1994), cyan (Heim and Tsien, 1996), yellow (Ormo et al., 1996), and red (Mishin et al., 2008) fluorescent proteins. Of these, the blue, cyan, and yellow variants (“BCaMP,” “CyCaMP,” and “YCaMP,” respectively) displayed spectral shifts comparable to the GFP variants and were optimized by subsequent mutagenesis. However, the red variant of GCaMP3 did not display any fluorescence and this variant was not pursued further. To create a red GECI, we selected the red fluorescent protein (FP) mRuby (an engineered variant of eqFP611 exhibiting high thermodynamic stability and monomericity) (Kredel et al., 2009) and replaced cpEGFP with circularly permuted mRuby in the GCaMP3 scaffold. Subsequent systematic engineering produced a red GECI (“RCaMP”). High-resolution crystal structures of mRuby in several states, as well as a structure of a Ca^2+^-loaded RCaMP variant, aided optimization of the red GECI scaffold to useful performance levels. Furthermore, the high-resolution structure of Ca^2+^-loaded R-GECO1 explains mutagenesis data and presents the opportunity for structure-guided optimization of this sensor as well.

We thoroughly characterize the new RCaMPs, both under single- and two-photon illumination, and present a number of novel applications. We demonstrate 2-color red/green calcium imaging both intra-cellularly (mitochondria and cytoplasm) and inter-cellularly (neurons and astrocytes). We show *in vivo* RCaMP imaging in worms, fly larvae, and zebrafish. We demonstrate integrated optogenetics experiments with RCaMP and ChR2, both in cells and *in vivo* in partially restrained worms, and make a comparison with GCaMP and the red-shifted opsin C1V1 (Yizhar et al., 2011b), and to recently published (but potentially artifactual) use of ChR2 with R-GECO1 and its mutants (Chang et al., 2012; Ohkura et al., 2012). Finally, we present a thorough spectral characterization of RCaMP and R-GECO1 focused on multicolor/optogenetics implementation. We compare two-photon bleaching of RCaMP and mRuby to the red Ca^2+^ sensor R-GECO1 (Zhao et al., 2011a).

We find that R-GECO1 shows dramatic reversible photoactivation and fast multi-state photobleaching that complicate implementation of the sensor, whereas RCaMP sensors are spectrally pure, brighter than R-GECO1 under 2-photon excitation and show no photoswitching. The new RCaMP sensors are the only currently available reagents for artifact-free, simultaneous optogenetics and functional imaging, and as such enable a host of qualitatively new experiments.

## Results

### Structure-guided engineering of color-shifted GECIs

**Figure 1:**
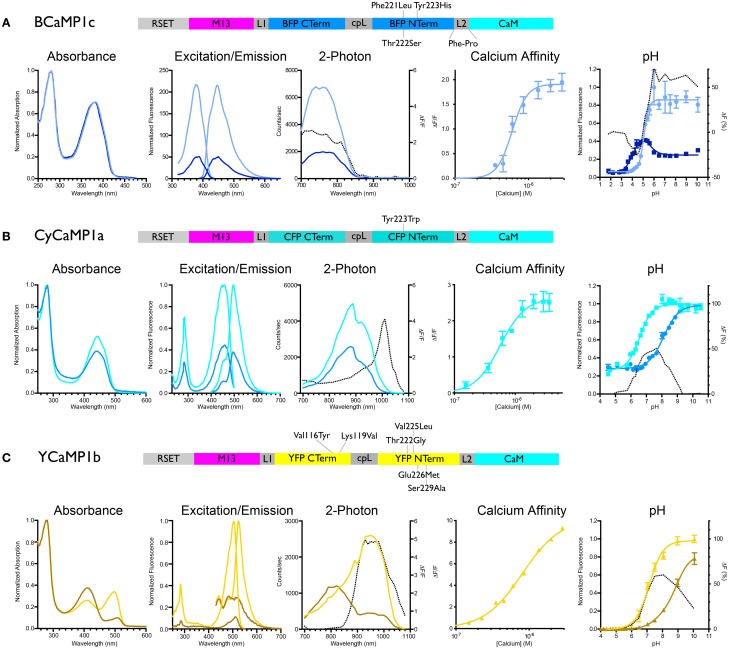
**Biochemical characterization of BCaMP, CyCaMP, and YCaMP.** Schematic for each color variant is given. For all variants, the lighter-colored lines indicate calcium-saturated spectra, darker-colored lines indicate calcium-free spectra. The dotted line in the 2-photon graph indicates Δ*F*/*F*, in the pH graph it indicates the difference in fluorescence (%). **(A)** BCaMP1c, **(B)** CyCaMP1a, **(C)** YCaMP1b.

We initially mutated GCaMP3 (Tian et al., 2009) to incorporate sets of the core mutations of EBFP, ECFP, and EYFP: in GCaMP3 numbering Phe221Leu/Thr222Ser/Tyr223His (“BCaMP1a”); Tyr223Trp (“CyCaMP1a”); and Val116Tyr/Lys119Val (GCaMP3 incorporates the Thr116Val mutation; “YCaMP1a”). CyCaMP1a and YCaMP1a were fluorescent and responded to *in vitro* calcium changes [(Δ*F*/*F*)_max_ = 2.6 ± 0.1, s.d., *n* = 3; and (Δ*F*/*F*)_max_ = 3.0 ± 0.1, s.d., *n* = 3, respectively] (Table 1), whereas BCaMP1a was fluorescent but not a sensor. The Cerulean (Rizzo et al., 2004) mutation Ser229Ala (GFP numbering Ser72Ala; Cerulean also has mutations at GFP positions 145 and 148, but these positions are absent in GCaMP) resulted in higher apo brightness and lower (Δ*F*/*F*)_max_ (1.9 ± 0.3, s.d., *n* = 3) in CyCaMP1b (Table 1, Figure 1B). YCaMP1a was improved by the incorporation of the Citrine (Griesbeck et al., 2001) mutations Thr65/222Gly, Val68/225Leu, Gln69/226Met, and Ser72/229Ala, resulting in YCaMP1b [(Δ*F*/*F*)_max_ = 9.2 ± 0.4, s.d., *n* = 3] (Table 1, Figure 1C). We incorporated the EBFP2 (Sato et al., 2007) and Azurite (Mena et al., 2006) mutations into BCaMP1a, but these did not result in improved variants. Screening variants of the M13pep-cpFP linker (“linker1”) produced a variant BCaMP1b, with amino acids Leu-Glu replaced by Met-Pro, with (Δ*F*/*F*)_max_ of 0.9 ± 0.2 (s.d., *n* = 3; Table 1). A cpFP-CaM linker (“linker2”) variant BCaMP1c with Phe-Pro instead of Thr-Arg, had a (Δ*F*/*F*)_max_ of 2.0 ± 0.1 (s.d., *n* = 3; Table 1, Figure 1A). The combination of the two linker variants resulted in a poor sensor (data not shown).

**Table 1:** **Biophysical characteristics of GECIs**.

**GECI variant**	**Mutations^a^**	**Max. *in vitro* Δ*F*/*F***	***F*_base_, *F*_max_ HEK^c^**	**Ratio (***F***_max_/*F*_base_) HEK**	**Baseline brightness cultured neurons**	**Ca^2+^ affinity (*K*_d_)**	**pK_a_ (sat/apo)**	**Hill coefficient**
GCaMP3	–	12.3 ± 0.4	–	–	–	405 ± 9 nM	6.97 ± 0.01/8.40 ± 0.02	2.1 ± 0.1
BCaMP1a	F221L, T222S, Y223H	–	–	–	–	–	–	–
BCaMP1b	L59M, E60P, F221L, T222S, Y223H	0.9 ± 0.2	–	–	–	958 ± 156 nM	5.3 ± 0.1/4.0 ± 0.16	1.7 ± 0.1
BCaMP1c	F221L, T222S, Y223H, T302F, R303P	2.0 ± 0.1	–	–	–	599 ± 26 nM	5.1 ± 0.1/4.2 ± 0.1 and 5.2 ± 0.26	2.7 ± 0.2
YCaMP1a	V116Y, K119V	3.0 ± 0.1	–	–	–	351 ± 24 nM	7.96 ± 0.02/8.64 ± 0.02	1.6 ± 0.1
YCaMP1b	V116Y, K119V, T222G; V225L; Q226M, S229A	9.2 ± 0.4	–	–	–	809 ± 46 nM	7.14 ± 0.02/8.9 ± 0.02	1.7 ± 0.1
CyCaMP1a	Y223W	2.6 ± 0.1	–	–	–	421 ± 41 nM	6.56 ± 0.04/8.12 ± 0.03	2.1 ± 0.3
CyCaMP1b	Y223W, S229A	1.9 ± 0.3	–	–	–	543 ± 37 nM	6.9 ± 0.3/7.55 ± 0.2	1.8 ± 0.2
RCaMP-AI	L59A, E60I, D114S	2.6 ± 0.1	Not detectable	–	–	3.8 ± 0.03 μ M	–	1.4 ± 0.2
RCaMP1a	L59A, E60I, A112Y, D114S, T364I	7.2 ± 0.1	1 ± 0.15; 1.4 ± 0.18	1.4 ± 0.1	–	1.3 ± 0.04 μ M	–	2.9 ± 0.1
RCaMP1b	L59A, E60I, G109A, A112Y, D114S, T364I	4.2 ± 0.1	–		–	770 ± 55 nM	–	2.6 ± 0.1
RCaMP1c	L59A, E60I, G109A, A112Y, D114S, T364I, D372Y	7.5 ± 0.1	0.76 ± 0.1; 1.1 ± 0.14	1.5 ± 0.1	–	922 ± 34 nM	5.03 ± 0.04/4.9 ± 0.2 and 6.9 ± 0.3	2.6 ± 0.1
RCaMP1d	L59A, E60I, G109A, A112Y, A270V, T364I, D372Y	6.5 ± 0.2	0.79 ± 0.11; 1.2 ± 0.13	1.5 ± 0.13	7.8 ± 0.2	1.6 ± 0.06 μ M	5.9 ± 0.1/4.8 ± 0.1	3.4 ± 0.4
RCaMP1e^b^	L59A, E60I, G109A, A112Y, D114S, A270V, T364I, D372Y	–	0.67 ± 0.06; 0.86 ± 0.1	1.3 ± 0.08	1. 0 ± 0.02	–	–	–
RCaMP1f^d^	L59A, E60I, G109A, A112Y, A270V, T364I, D372Y, R384G, I424S	12.3 ± 0.2	1.46 ± 0.08; 2.6 ± 0.2	1.8 ± 0.2	6.7 ± 0.1	1.9 ± 0.08 μ M	5.32 ± 0.02/4.6 ± 0.1	2.8 ± 0.4
RCaMP1h	L59A, E60I, G109A, A112Y, A270V, R295D, D296S, T364I, D372Y, R384G, I424S	10.5 ± 0.1	1.6 ± 0.1; 3.2 ± 0.26	2.0 ± 0.1	8.3 ± 0.1	1.3 ± 0.06 μ M	4.94 ± 0.05/~3.2, ~4.7 and ~6.5^e^	2.5 ± 0.1
R-GECO1	–	10.5 ± 0.1	1.22 ± 0.11; 3.07 ± 0.32	2.5 ± 0.5	1.02 ± 0.04	449 ± 21 nM	6.59 and 8.9^f^	1.51 ± 0.1 0.03

a *BCaMP, YCaMP, and CyCaMPs also contain the mutations R2 deletion, M65K, T115V, and N362D, as in GCaMP3*.

b *RCaMP1e is identical to RCaMP1d, but without the RSET tag. Numbering is kept in line with RCaMP-AI*.

c *Cultured neuron baseline brightness is normalized to the brightness of RCaMP1e*.

d *RCaMP1f lacks 2 histidines in the RSET—nickel affinity tag. This is not taken into account in the numbering*.

e *The pH titration of RCaMP1h (apo) is quite complicated; values are estimated rather than determined from curve fitting*.

f *Data from Zhao et al. (2011a)*.

### Initial engineering of the RCaMP scaffold

**Figure 2:**
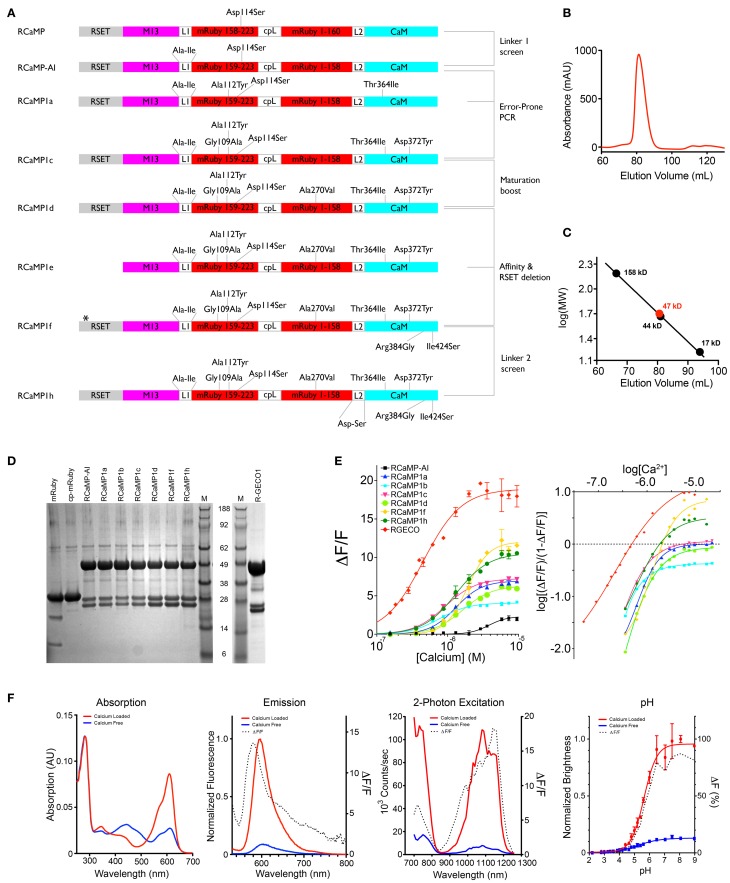
**RCaMP engineering and *in vitro* characterization. (A)** Schematic of the RCaMP design process. The star indicates a deletion of two histidines in the poly-histidine tag in RCaMP1f. Numbering is based on RCaMP-AI. **(B)** Size exclusion chromatogram of purified Ca^2+^-RCaMP1h. **(C)** Calibration curve calculated from molecular weight standards and the estimated mass of RCaMP (47 kD) based on elution volume (bottom). The calculated mass of RCaMP is ~49.2 kD, indicating that RCaMP exists primarily as a monomer in solution. **(D)** SDS-PAGE of mRuby, RCaMP variants, and R-GECO1. Lane M indicates molecular size marker SeeBlue Plus 2 (Invitrogen). The calculated mass of RCaMP is 49.2 kD (for R-GECO1, 46.9 kD), and the fragments resulting from imine hydrolysis at the chromophore are calculated as 23.2 and 25.9 kD (for R-GECO1, 20.8 and 26.1 kD). For mRuby the calculated masses are 29.3 kD for full-length and 10.9 kD and 18.4 kD for the putative fragments (calculated with AphaEase FC, Genetic Technologies Inc., USA). **(E)** Ca^2+^ titrations of purified protein (left). Right: Hill plot of data shown left with linear range of sensors at lower calcium concentrations. **(F)** *In vitro* spectroscopic analysis of RCaMP1f (from left to right): Absorption, emission, 2-photon spectra, and pH titrations. 2-photon spectra were acquired from 700–1080 nm with Ti: Sapphire illumination, and from 1080–1260 nm with OPO illumination.

A red-colored (albeit quite dim) variant of *Aequorea victoria* GFP has been published (Mishin et al., 2008); we made the corresponding mutations to GCaMP3, but the resulting protein displayed no fluorescence (data not shown). Subsequently, we circularly permuted the red FP mCherry (Shaner et al., 2004) and replaced cpGFP in GCaMP3 with cp-mCherry; this protein was not fluorescent either (data not shown), in agreement with the absence of chromophore formation in cp-mCherry described previously (Carlson et al., 2010). We therefore selected another protein, mRuby (Kredel et al., 2009), as a template for a red calcium indicator. We reasoned mRuby would be more amenable to circular permutation because of its robust thermodynamic stability. Its parental protein, eqFP611, has been shown to contain a *trans*-conformation of its chromophore (Petersen et al., 2003), shifting the tyrosyl moiety of the chromophore in between β-strands 7 and 8 of the FP barrel. Interestingly, mutagenesis of eqFP611 to RFP639 shifts the chromophore to the *cis*-conformation (Nienhaus et al., 2008), bringing the tyrosyl moiety in close proximity to strand 10 as well. We therefore tried two different circular permutations, at residues 159 (strand 7) and 196 (strand 10), and additionally tried swapping the M13pep and CaM domains in each circular permutation (see Figure 2A for a schematic of the RCaMP family). The RCaMP scaffold with the circular permutation at the same region as GCaMP (after amino acid 159), sandwiched between N-terminal M13pep and C-terminal CaM in the same positions, was very dimly fluorescent in *Escherichia coli* colonies after prolonged (4 days) incubation at 4°C; the other scaffolds were not fluorescent (not shown) and abandoned during further optimization.

Random mutagenesis of linker1 (M13pep-to-cp-mRuby; originally the same Leu59-Glu60 as in GCaMP, from the translated XhoI site) resulted in four variants (Pro-Arg-Ile, Cys-Ile, Arg-Ile, Ala-Ile) with faster onset of fluorescence in *E. coli* (~1 day). The variants expressed well in *E. coli* and eluted as monomers during size exclusion chromatography (SEC) (Figures 2B,C). Denaturing SDS-PAGE gels showed that the RCaMPs exhibit a significant population of backbone-cleaved species (Figure 2D). Of the four variants, the Ala-Ile linker variant (“RCaMP-AI”) showed the largest (Δ*F*/*F*)_max_ (2.6 ± 0.1, s.d., *n* = 3) (Table 1, Figure 2E) and was selected for further optimization. A spontaneous mutation that arose during gene assembly (Asp114Ser) was also incorporated into RCaMP (Table 1). Fluorescence spectra of purified RCaMP-AI with 450 nm excitation indicated the presence of a substantial green fluorescent component (not shown), potentially from incomplete maturation of the red species by stalling in a green intermediate state, as proposed for the red FP zFP574 (Ivashkin et al., 2011). To optimize maturation, we performed error-prone PCR of the RCaMP-AI template and screened *E. coli* colonies for variants with faster and more complete red chromophore maturation. This produced RCaMP1a, with the additional mutations Ala112Tyr and Thr364Ile (Table 1).

### Crystal structures of mRuby, RCaMP, and R-GECO1

To aid our protein engineering efforts, we sought to solve the crystal structures of mRuby, RCaMP, and R-GECO1. Structural information on the parent protein of mRuby, eqFP611 (86% sequence identity with mRuby), has been published (Petersen et al., 2003), however, no protein structure of mRuby was available at the time of initial RCaMP engineering, hampering optimization efforts. It has previously been shown that pH can have a large effect on chromophore conformation in red FPs (Battad et al., 2007; Pletnev et al., 2008; Pletneva et al., 2011); therefore we determined the crystal structure of mRuby at two different pHs: 4.5 and 8.5 (Table 2). The large difference in pH had no notable effect on overall mRuby protein structure (RMSD = 0.13 Å for 208 C_α_ atoms). The structure of mRuby at pH 8.5 shows a *trans*-isomer of the red 2-(iminomethyl)-4-(4-hydroxybenzylidene)-imidazol-5-one chromophore, while the structure at pH 4.5 shows a mixture of ~60% *trans* and ~40% *cis*. Similar pH-dependent *cis-trans* conformational changes also occur in the related FP eqFP578 (Pletneva et al., 2011) (70% sequence identity with mRuby). For other RFPs [mKate (Pletnev et al., 2008), Katushka (Pletneva et al., 2011) and RtmS5-H148S (Battad et al., 2007); 68, 67, and 51% sequence identity with mRuby, respectively], an opposite pH-induced isomerization of the chromophore has been described. The RMSD between mRuby and eqFP611 is 1.11 Å for 195 common C_α_ atoms, with the largest differences in surface loops resulting from different crystal packing. It was previously shown that the equilibrium of *cis-trans* isomers of the chromophore in eqFP611 can be influenced by altering the hydrogen-bonding network surrounding the chromophore (Nienhaus et al., 2008), further complicating the predictability of RCaMP chromophore behavior. To directly observe the chromophore orientation and the structure and extent of cp-mRuby/CaM packing interactions, both of which assisted GCaMP engineering (Akerboom et al., 2009, 2012a; Tian et al., 2009), we solved the crystal structure of the early RCaMP variant RCaMP1a.

**Table 2:** **X-ray diffraction data collection and model refinement statistics**.

	**mRuby 1 pH 4.5 (PDB 3U0L)**	**mRuby 1 pH 8.5 (PDB 3U0M)**	**mRuby 2 (PDB 3U0N)**	**Ca^2^+-RCaMP (PDB 3U0K)**	**Ca^2^+-RGECO1 (PDB 4I2Y)**
**DATA COLLECTION**					
Space group	P2_1_2_1_2_1_	P2_1_2_1_2_1_	I222	P3_2_21	P2_1_
Cell dimensions					
a (Å)	31.77	31.71	67.27	75.92	65.16
b (Å)	67.44	67.08	82.52	75.92	91.73
c (Å)	95.06	95.00	88.91	123.16	68.57
β (degrees)	90	90	90	120	95.3
Beamline	APS 31-ID	APS 31-ID	APS 31-ID	APS 31-ID	ALS 8.2.2
Wavelength (Å)	0.9793	0.9793	0.9793	0.9793	1.000
Resolution range (Å)^a^	19.43 − 1.25	28.66 − 1.65	27.90 − 1.60	34.82 − 2.10	54.77 − 2.20
Total reflections	595,508	256,831	277,856	404,612	151,067
Unique reflections	56,036	24,632	32,955	24,607	40,865
Completeness (%)	97.8 (95.8)	97.9 (96.9)	99.8 (100)	99.9 (100)	100 (100)
I/σ^a^	16.5 (4.6)	19.7 (4.6)	11.7 (4.7)	20.0 (6.7)	8.8 (2.6)
*R*_sym_ (%)^a^^,^^b^	7.3 (56.2)	7.1 (58.4)	12.8 (51.3)	10.3 (54.5)	10.5 (58.8)
**REFINEMENT**					
*R*_work_/*R*_free_ (%)^c^	12.6/16.1	17.2/20.1	15.4/17.9	18.9/23.9	19.4/25.7
Resolution range (Å)	20 − 1.25	30 − 1.65	30 − 1.60	22 − 2.10	54 − 2.20
Number of atoms (B factor)					
protein	1957 (14.4)	1823 (19.7)	1854 (12.5)	3160 (15.6)	6092 (30.9)
water	231 (28.7)	146 (28.9)	195 (26.2)	133 (33.4)	99 (27.0)
other	4 (20.3)	–	10 (31.1)	8 (41.0)	8 (27.6)
RMSD values					
Bond lengths (Å)	0.028	0.024	0.031	0.022	0.021
Bond angles (degrees)	2.48	2.24	2.63	1.90	1.91
Ramachandran (%)					
Favored/disallowed	98.6/0	98.6/0	99.1/0	96.7/0.8	97.9/0.4
Molprobity					
Clashscore (percentile)	14.73 (17)	7.37 (85)	9.57 (67)	10.4 (84)	13.21 (79)
Molprobity score (percentile)	1.67 (60)	1.57 (87)	1.53 (87)	2.08 (75)	2.31 (66)

a *The number in parentheses is for the highest resolution shell*.

b *R_sym_ = Σ^i^_hkl_|I^i^_(hkl)_ – <I_(hkl)_>| / Σ_hkl_ <I_(hkl)_>, where I^i^_(hkl)_ is the ith measured diffraction intensity and <I_(hkl)_> is the mean of the intensity for the miller index (hkl)*.

c *R_work_ = Σ_hkl_|| F_o_(hkl)| – |F_c_(hkl)|| / Σ_hkl_ |F_o_(hkl)|. R_free_ = R_work_ for 5% of reflections not included in refinement*.

**Figure 3:**
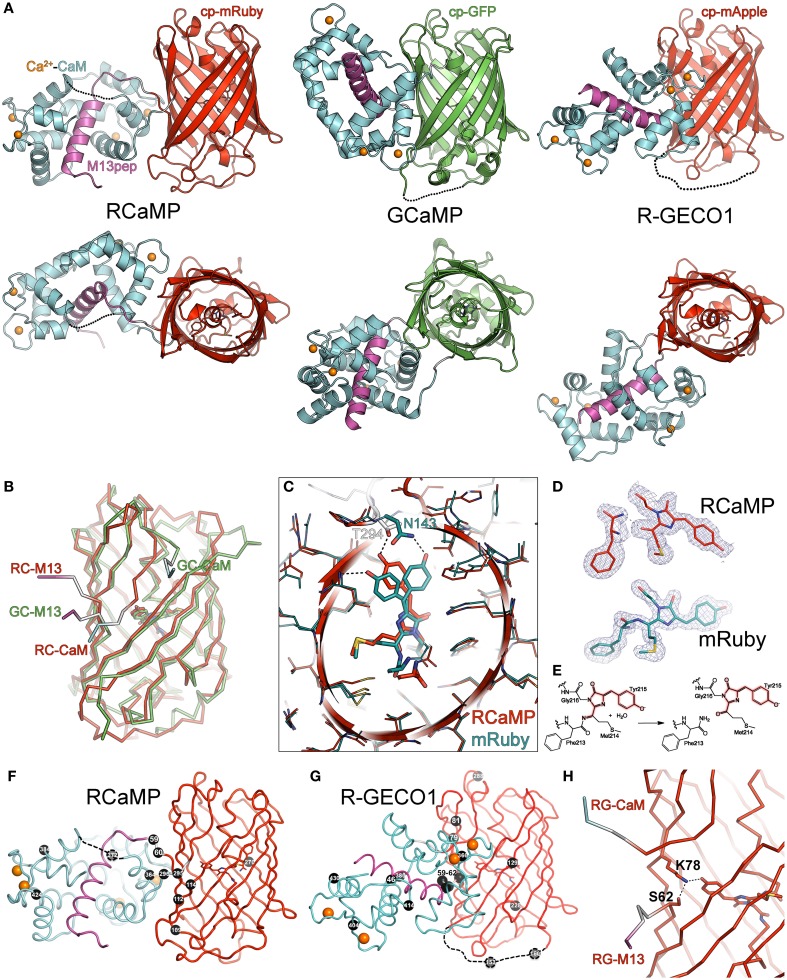
**Crystal structures of RCaMP, mRuby, and R-GECO1. (A)** Crystal structures of Ca^2+^-bound RCaMP, GCaMP, and R-GECO1 in two orthogonal views. The fluorescent protein domain of each sensor was superimposed. Labels indicate the coloring of the domains of RCaMP; coloring of GCaMP and R-GECO1 is identical except that the cpGFP domain is colored green. **(B)** Ribbon diagram of the superimposed circularly permuted fluorescent protein domains of RCaMP and GCaMP illustrating structural differences at the circular permutation site. Coloring is the same as in **(A)**. Linker connections to the M13 peptide and CaM domains are labeled. **(C)** Superposition of the cp-mRuby domain of RCaMP with mRuby. Amino acids are displayed as thin sticks, except the chromophore and select chromophore-interacting residues, which are shown as thicker sticks. RCaMP is additionally displayed as a cartoon, colored as in **(A)**. mRuby is colored cyan. Select hydrogen bonds are displayed as dashed lines. View is oriented and clipped to show the center of the fluorescent protein barrels. **(D)** The chromophores and preceding amino acid of RCaMP (top, red) and mRuby (bottom, cyan) with the 2Fo-Fc omit electron density map contoured at 1σ superimposed. Note the absence of electron density in RCaMP, indicating backbone cleavage. **(E)** Proposed hydrolysis of the peptide bond in RCaMP. Atoms involved in the extended π-system of the fluorescent chromophore in red. **(F)** Ribbon depiction of RCaMP with sites of engineering shown as black spheres, with corresponding amino acid number shown. Orientation is as shown for **(A)**. **(G)** As in **(F)**, but for R-GECO1. Depicted sites of engineering from Zhao et al. (2011a). **(H)** In Ca^2+^-bound R-GECO1, Lys78 forms an ionic hydrogen bond with the chromophore, stabilized by Ser62.

RCaMP1a was crystallized in the Ca^2+^-bound form and its structure was determined to 2.1 Å resolution by molecular replacement (Table 2). RCaMP crystallized as a monomer, consistent with solution measurements (Figures 3A, 2B,C).

Although the mRuby circular permutation was based on GCaMP, large differences between RCaMP and GCaMP in both the orientation of, and interface between, the CaM/M13 complex and the cpFP domain can be seen (Figures 3A,B). The CaM/M13 complex of Ca^2+^-RCaMP is generally similar to that of Ca^2+^-GCaMP (RMSD of 1.9 Å for 152 common C_α_ atoms), but adopts a slightly more closed and compact conformation. The cp-mRuby domain of RCaMP is structurally very similar to mRuby, with an RMSD of 0.5 Å for 209 C_α_ atoms. Strikingly, unlike GCaMP, there is no large opening in the side of the FP β-barrel created by the circular permutation of the mRuby domain in Ca^2+^-bound RCaMP (Figure 3B); the termini of cp-mRuby cross close to one another and produce minimal disruption to the β-barrel. In contrast to the construction of the original GCaMP protein (Nagai et al., 2001), no amino acids of mRuby were replaced by linker amino acids. As a consequence of the circular permutation, though, linker amino acids partake in β-barrel formation in the cp-mRuby domain of RCaMP1a: residue Asn143 of mRuby, which directly hydrogen bonds the phenolate oxygen of the chromophore, is spatially substituted by Thr294 of the second linker of RCaMP (Figure 3C); the crystal structure shows that the asparagine (Asn61 in RCaMP) is co-opted into the first linker. Mutagenesis of this threonine to any other amino acid, or deletion of amino acids to generate an opening similar to GCaMP, greatly diminishes fluorescence of RCaMP (data not shown).

In contrast with the chromophore in the structure of mRuby crystallized at pH 8.5, the RCaMP structure (crystallized at pH 7.5) shows the (4-hydroxybenzylidene)-imidazol-5-one moiety of the chromophore to be a *cis*-isomer (Figures 3C,D). Superimposition of both mRuby structures and the cp-mRuby domain of RCaMP demonstrates a slight (~1 Å) shift of the RCaMP chromophore (red structure in Figure 3C) relative to both isomers of the mRuby chromophore (cyan in Figure 3C). Closer inspection of the electron density map for the RCaMP chromophore revealed a discontinuity between Phe213 and Met214, which forms part of the chromophore (Met214-Tyr215-Gly216) (Figure 3D). The electron density map is consistent with imine hydrolysis of the RCaMP chromophore, resulting in a ketone on Met214, maintaining the extended π-conjugation of the red chromophore (thus 2-acyl-4-(4-hydroxybenzylidene)-imidazol-5-one), and an amide on Phe213 (Figures 3D,E). This imine hydrolysis results in a discontinuity in the polypeptide backbone of RCaMP, also observed by SDS-PAGE of purified protein samples (Figure 2D). R-GECO1 showed a comparable level of backbone cleavage, with two smaller protein bands corresponding to imine hydrolysis of the chromophore (Figure 2D). The electron density map for mRuby shows no significant imine hydrolysis (Figure 3D). However, after boiling in the presence of the reducing agent 2-mercaptoethanol during sample preparation for SDS-PAGE, cleavage products can clearly be detected (Figure 2D), in agreement with structural and SDS-PAGE analysis of the red FP DsRed (Gross et al., 2000). Equivalent amide hydrolysis is reported for the photoactivatable Kindling Fluorescent Protein (asFP595) (Tretyakova et al., 2007). Interestingly, cp-mRuby does not show significant levels of imine hydrolysis (Figure 2D), and also lacks chromophore formation (not shown). The red-shifting (~15 nm excitation, ~4 nm emission) of the RCaMP chromophore relative to mRuby may result from rearrangements around the chromophore, as seen for eqFP611, RFP611, RFP618, RFP630, and RFP639 (Kredel et al., 2008), or from planarization following chromophore hydrolysis.

In addition to mRuby and RCaMP, we also crystallized R-GECO1 (Zhao et al., 2011a) in the presence of Ca^2+^ and solved the crystal structure to 2.2 Å by molecular replacement (Table 2). Strikingly, the position of the CaM domain relative to cp-mApple is distinct from that seen in RCaMP and GCaMP (Figure 3A)—the homology model used during R-GECO1 design (Zhao et al., 2011a)—underlining the importance of structure determination in protein engineering. Many of the mutations incorporated into the R-GECO1 sensor during initial screening and selection (Zhao et al., 2011a) are concentrated at the proto-interface between mApple and CaM (Figure 3G), as is the case for RCaMP (Figure 3F). Three residues of the M13pep-cp-mApple linker (Val60, Val61, and Ser62) make hydrophobic and hydrogen-bonding contacts with CaM, cp-mApple, and the rest of the linker. Similar to GCaMP (Wang et al., 2008; Akerboom et al., 2009), the cp-mApple-CaM linker is not in close proximity to the chromophore, and no cp-mApple-CaM linker mutations were selected in R-GECO1 (Zhao et al., 2011a). In addition to the linkers, R-GECO1 mutations Gly79, Arg81, Cys129, Phe366, and Asn380 are in the vicinity of the proto-interface (Figure 3G). No backbone cleavage was observed in the vicinity of the R-GECO1 chromophore, as was seen in the RCaMP crystal structure. R-GECO-1-Lys78, from strand eight of cp-mApple, adjacent to the circular permutation site, forms an ionic interaction with the phenolate oxygen of the chromophore and is stabilized in place via a hydrogen bond from Ser62, immediately following the M13pep-cp-mApple linker (Figure 3H).

### Optimization of RCaMP

The structure of RCaMP explains the initial selection of mutations resulting in RCaMP1a, and allowed us to prioritize sites of mutagenesis for additional rounds of sensor engineering. As in GCaMP (Wang et al., 2008; Akerboom et al., 2009), the circular permutation of mRuby and fusion of M13pep and CaM resulted in a tightly packed adventitious interface between CaM and cp-mRuby, excluding solvent from the chromophore environment. The first linker, connecting M13pep and cp-mRuby, comes in close proximity to the chromophore, and the strongly selected isoleucine side-chain packs tightly against the inter-domain linker of CaM and cp-mRuby. Thr294, originally from the translated MluI restriction site in the second linker, hydrogen-bonds the RCaMP chromophore, stabilizing it in the deprotonated state (Figure 3C). The spontaneous (PCR-derived) Asp114Ser mutation improves packing against the second linker, especially Gln297 (Figure 3F).

Both mutations from error-prone PCR mutagenesis, Ala112Tyr and Thr364Ile, improve packing at the mRuby-CaM interface (Figure 3F). The improved packing of the interface by these mutations simultaneously increases brightness, red state maturation completeness and kinetics, (Δ*F*/*F*)_max_, affinity and cooperativity. A second round of mutagenesis selected Gly109Ala, further improving packing at the mRuby-CaM interface (Figure 3F) in RCaMP1b, with increased affinity at a slight loss in (Δ*F*/*F*)_max_ (Table 1). The GCaMP5 mutation CaM-Asp380Tyr (Akerboom et al., 2009) is positioned at the GFP-CaM interface near the GFP chromophore and led to an increase in the calcium-bound fluorescence of GCaMP (Akerboom et al., 2012a). The corresponding mutation to RCaMP1b, Asp372Tyr, gave rise to RCaMP1c, with (Δ*F*/*F*)_max_ = 7.5 (Table 1). RCaMP1d was discovered as a spontaneous, fast-maturing mutant; faster chromophore maturation comes at the cost of affinity (1.6 ± 0.06 μM, s.d., *n* = 3) and (Δ*F*/*F*)_max_ (6.5 ± 0.2, s.d., *n* = 3) (Table 1). RCaMP1d was found to contain the mutation Ala270Val; the corresponding mRuby mutation, Ala120Val, was independently observed recently during the generation of mRuby2 (Lam et al., 2012), which exhibits greater brightness and photostability. Deletion of the RSET peptide resulted in RCaMP1e, which was significantly dimmer in cells, perhaps indicating that RCaMP is stabilized *in situ* by this N-terminal fusion peptide, as were early GCaMP variants (Tallini et al., 2006). We attempted to increase affinity by introducing the CaM mutation Arg90Gly (Sorensen and Shea, 1996; Akerboom et al., 2012a) (RCaMP numbering Arg384Gly) and screening libraries around the four EF-hands of CaM, which produced RCaMP1f. Affinity surprisingly decreased slightly to 1.9 ± 0.08 μM (s.d., *n* = 3), but (Δ*F*/*F*)_max_ rose to 12.3 ± 0.2 (s.d., *n* = 3; Table 1, Figures 2A,E). Lastly, targeted mutagenesis of the second linker (cp-mRuby-to-CaM; encompassing the Thr294-Arg295 linker, as in GCaMP, from the translated MluI site, as well as the subsequent residue Asp296) resulted in RCaMP1h, with 1.3 ± 0.06 μ M affinity, and (Δ*F*/*F*)_max_ of 10.5 ± 0.1 (s.d., *n* = 3; Table 1, Figure 2A). The crystal structure of RCaMP1a suggests that the charge-reversal mutation Arg295Asp likely improves interactions with Arg116 from cp-mRuby (Figure 3F). (RCaMP1g did not perform well in initial characterization and was not pursued further).

Fluorescence response to Ca^2+^ titrations of purified RCaMP and R-GECO1 sensor proteins are shown in Figure 2E-left; a Hill plot of the data shows linear ranges (Figure 2E-right). All RCaMP variants after RCaMP-AI show near complete maturation to the red state and similar extent of backbone cleavage (Figure 2D). All RCaMPs behave as monomers in solution (Figures 2B,C) and are fluorescent indicators of Ca^2+^ under both 1- and 2- photon excitation (RCaMP1f is shown in Figure 2F).

### Photophysical characterization of chromatic variants

Next we purified several RCaMP variants, as well as R-GECO1 and the parent fluorescence proteins mRuby and mApple, and subjected them to systematic photophysical characterization, under both 1- and 2-photon illumination. We have previously analyzed the fluorescence mechanism of the GCaMP calcium indicator (Akerboom et al., 2009; Mütze et al., 2012), which involves a Ca^2+^-dependent deprotonation of the *p*-hydroxybenzylideneimidazolinone chromophore, leading to increased fluorescence. In GCaMP, the quantum yield (QY; and fluorescence lifetime) of the Ca^2+^-bound (bright) and Ca^2+^-free (dim) states are identical; the fluorescence increase upon Ca^2+^ binding is manifested entirely as an increase in extinction coefficient (ε), reflecting a greater population of deprotonated, fluorescent chromophore (Mütze et al., 2012). Strikingly, in the RCaMP indicators we found that more of the Ca^2+^-dependent increase in brightness was attributable to changes in quantum yield and fluorescence lifetime, than to changes in the extinction coefficient (Table 3). The peak of the RCaMP Ca^2+^-bound state is slightly spectrally shifted relative to the unbound state (from ~8 nm blue-shifted to ~1 nm red-shifted, for the different RCaMP sensors). R-GECO1 shows a larger peak shift: 12 nm blue-shifting of absorption, and 10 nm blue-shifting of emission. Under 1-photon illumination, the parent protein mRuby is slightly brighter (brightness = ε × QY) than EGFP; the brightness of the Ca^2+^-bound state of the different RCaMP indicators is 66–86% that of mRuby (Table 3).

**Table 3:** **Photophysical parameters of RCaMPs, R-GECO1, and parent fluorescent proteins**.

	**One-photon excitation**										**Two-photon excitation**				
	**λ_abs_ (λ_em_), nm**		**ε_peak_ (mM^−1^cm^−1^)**		**Quantum yield**		**Brightness, % (relative to EGFP)**		**Δ*F*/*F* 570ex, all em**.		**Δ*F*/*F* 1070ex, all em**.		**λ_abs_ (nm)**		**Peak brightness, 1070 nm (kcpsm)**	
	**+Ca^2^+**	**−Ca^2^+**	**+Ca^2^+**	**−Ca^2^+**	**+Ca^2^+**	**−Ca^2^+**	**+Ca^2^+**	**−Ca^2^+**					**+Ca^2^+**	**−Ca^2^+**	**+Ca^2^+**	
mRuby	558(590)	558(590)	94.6		0.41		113						1060		4.0	
RCaMP1a	576.5(595)	575.5(594)	56.7	34.8	0.45	0.12	75	12	4.1		4.3		1070	1070	5.9	
RCaMP1c	576.5(595)	576.5(597)	64.8	39.7	0.48	0.10	91	11.6	5.9		7.1		1070	1070	7.3	
RCaMP1d	572(592.5)	575(597.5)	57.8	25.4	0.52	0.13	88	9.6	8.7		11.1		1070	1070	7.7	
RCaMP1f	572(591.5)	574(597)	58.9	17.4	0.48	0.11	83	5.6	12.3		8.8		1070	1070	8.2	
RCaMP1h	571(594)	575(602)	65.1	18.7	0.51	0.14	97	7.7	10.0		10.9		1070	1070	8.1	
mApple	568(592)	568(592)	57.3		0.42		70						1070		7.0	
R-GECO1	564 (588)	576(598)	59.3	6.1	0.19	0.13	33	2.3	12.6		18.0		1065	1065	3.8	

pH titrations of the new sensors show some complex effects; some blue and red GECIs exhibit multiple titratable groups affecting fluorescence (Figures 1, 2, Table 1). Intriguingly, for some sensors, the pK_a_ of the Ca^2+^-bound state is higher than that of the Ca^2+^-free state (unlike GCaMP), implying that calcium binding decreases the acidity of the chromophore proton, increasing the protonated, and potentially non-fluorescent state of the chromophore (Tables 1, 3). The fact that the sensors nonetheless exhibit such large increases in fluorescence implies that the photophysical transitions upon calcium binding are more complicated than in GCaMP, consistent with the Ca^2+^-dependent increase in quantum yield, and the pH-dependent *cis*-*trans* conformational changes seen in RCaMP and other RFPs (Battad et al., 2007; Pletnev et al., 2008; Pletneva et al., 2011). R-GECO1, by contrast, shows a GCaMP-like Ca^2+^-dependent decrease of pK_a_ from 8.9 to 6.6 (Zhao et al., 2011a), consistent with a fluorescence increase driven largely by extinction coefficient effects (Table 3).

Importantly, RCaMP shows large (Δ*F*/*F*)_max_ signals under 2-photon illumination, on the order of the 1-photon signal change (Figure 2F). Under 2-photon illumination, the RCaMP sensors are red-shifted and surprisingly brighter than mRuby (Table 3). For the red indicators, excitation with either a Ti:Sapphire laser or an optical parametric oscillator (OPO) coupled to a primary exciplex laser produced large fluorescence changes upon calcium addition (Figure 2F).

**Figure 4:**
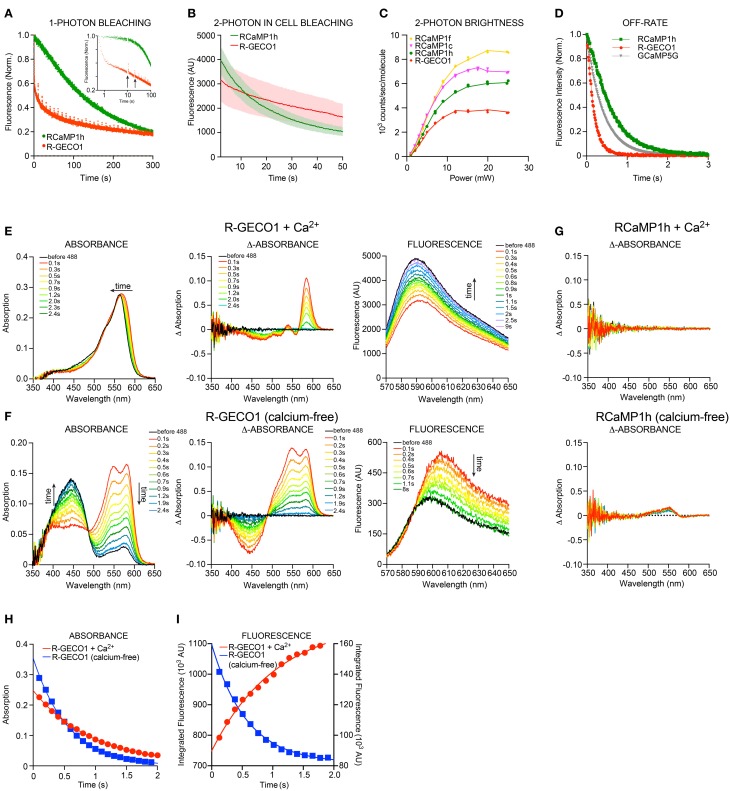
**Biophysical characterization RCaMP and R-GECO1. (A)** 1-photon Photobleaching of RCaMP1h and R-GECO1 compared. Note the fast decay during the first second and partial re-activation of R-GECO1 during darkness (after each 10 s) of R-GECO1 (arrows). **(B)** 2-photon photobleaching of RCaMP1h and R-GECO1 in HEK293 cells. **(C)** 2-photon peak brightness spectra (Mütze et al., 2012) of select RCaMPs and R-GECO1 compared. **(D)** Stopped-flow fluorescence of R-GECO1, GCaMP5G, and RCaMP1h. Protein concentration, 1 μM. Initial [Ca^2+^], 10 μM. [EGTA], 10 mM. **(E,F)** Transient response of 10 μ M R-GECO1 following a pulse of 488-nm light at 600 mW/cm^2^ for 1 s, in +Ca^2+^ buffer **(D)** and Ca^2+^-free buffer **(E)**, showing time evolution of absolute absorbance (left panels), transient absorbance pre-488 and post-488 (middle panels), and absolute fluorescence induced by weak 561-nm excitation before and following the 488-nm pulse (right panels). **(G)** In contrast with **(D)** and **(E)**, 10 μM Ca^2+^-free RCaMP1h shows only a very small increase in absorbance around 550 nm (lower panel) and no change in absorbance in +Ca^2+^ buffer (upper panel). No differences are seen in fluorescence (data not shown). **(H)** The transient absorption decay in time is well fit by exponential decay kinetics with e^-1^ time constants of 0.96 s and 0.58 s for +Ca^2+^ and no-Ca^2+^, respectively. **(I)** R-GECO1 transient fluorescence is also well fit by exponential decay kinetics with e^-1^ time constants of 1.01 s and 0.56 s for +Ca^2+^ buffer and no-Ca^2+^ buffer, respectively. Note that the transient fluorescence increases following the 488-nm pulse in no-Ca^2+^ buffer, but decreases in +Ca^2+^ buffer.

Red FPs frequently exhibit poor photostability (Drobizhev et al., 2011); this is a serious drawback that limits their utility in experiments. Other photophysical phenomena also complicate usage; for instance, mApple shows significant photoswitching (Shaner et al., 2008). We examined the photoswitching and photobleaching properties of the red GECIs and their parent FPs under 1-photon (either lamp excitation or wide-field laser illumination) and 2-photon (laser) regimes. As published, mApple showed an immediate, ~50% drop in fluorescence upon illumination, which recovered slowly during darkness (data not shown). R-GECO1 showed similar rapid-photoswitching properties (Figure 4A), which may confound functional imaging. RCaMP1h displayed a more conventional, irreversible form of photobleaching, and appeared to bleach more slowly for the first several minutes of bright mercury lamp illumination (2.7 W/cm^2^). Two-photon illumination of HEK293 cells expressing RCaMP1h or R-GECO1 showed a somewhat faster decay of fluorescence for RCaMP1h compared to R-GECO1 (Figure 4B). However, 2-photon brightness of several RCaMPs was more than double that of R-GECO1 (Table 3, Figure 4C). Stopped-flow fluorescence showed that decay kinetics of R-GECO1 are about twice as fast as GCaMP5G; RCaMP1h is slightly slower than GCaMP5G (t_1/2_(decay): R-GECO1, 120 ms; GCaMP5G, 280 ms; RCaMP1h, 410 ms) (Figure 4D).

Strikingly, R-GECO1 displayed significant photoactivation after illumination (~2-fold) with 405 nm, 488 nm and 561 nm light for both calcium-free and calcium-loaded states of the protein, although the activation is different in nature between the two states. This effect was strongest for 488 nm, followed by 405 nm and approximately 10-fold less for 561 nm. Applying 1 s pulses of 488 nm light resulted in a red-shift in absorbance of calcium-loaded R-GECO1 (Figure 4E-left), resulting in a temporary decrease (~40%) in fluorescence when illuminated with 561 nm (Figure 4E-right). For calcium-free R-GECO1, 1 s pulses of 488 nm light resulted in an apparent decrease in absorbance of protonated chromophore and a simultaneous increase in absorbance in deprotonated chromophore (Figure 4F-left), mimicking the effect on fluorescence of calcium binding to R-GECO1 (Figure 4F-right) when illuminated with 561 nm light. This effect resulted in an apparent increase in fluorescence (~50%, Figure 4F-right). Photo-activation could be observed using a range of illumination intensities (0.056–0.66 mW/cm^2^, 488 nm, not shown). Transient decay of the photoactivated state for both calcium-free and calcium-loaded R-GECO1 followed one-phase decay kinetics, with a half-life of 560 ms and 940 ms, respectively (Figures 4H,I). There is also a wavelength shift in the transient fluorescence, a small red-shift of 2 nm for Ca^2+^-bound and 7.5 nm for Ca^2+^-free.

These photophysical effects are not observed for RCaMP (Figure 4G), suggesting that RCaMP may be a better sensor to be used in combination with optogenetic tools.

### Characterization in HEK293 cells and neurons

**Figure 5:**
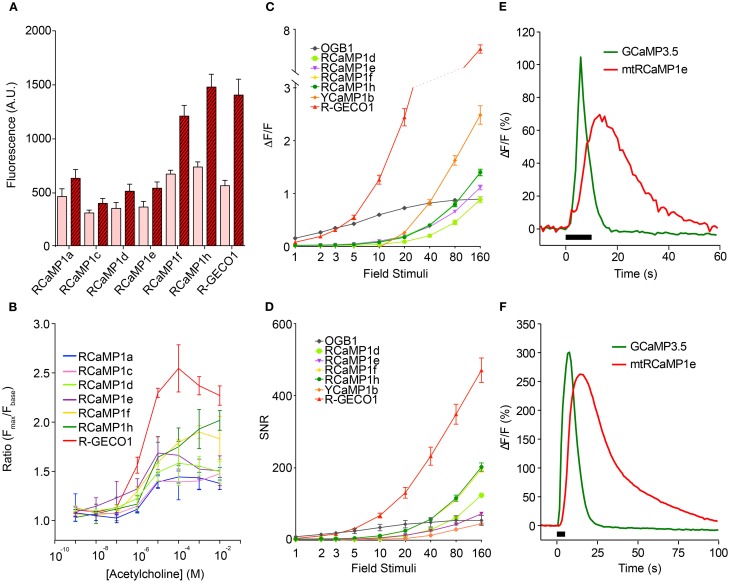
**Characterization of the RCaMP, R-GECO1, and YCaMP in cells. (A)** Baseline and peak fluorescence of HEK293 cells expressing the red GECIs, in response to acetylcholine (Ach)-induced Ca^2+^ mobilization. **(B)** Fluorescence response (*F*_max_/*F*_baseline_) for the red GECIs vs. Ach concentration. **(C)** Fluorescence increase (Δ*F*/*F*)_max_ of the red and yellow GECIs, as well as OGB-1, in cultured rat hippocampal neurons following electrode-evoked action potential trains. Plotted is mean ± sem (*n* = 13–16). **(D)** Signal-to-noise ratio of the data shown in **(C)**. Plotted is mean ± sem (*n* = 13–16). **(E,F)** Δ*F*/*F* time-lapse traces from rat cortical neurons expressing RCaMP1e in mitochondria (red) and GCaMP3.5 in cytosol (green) after caffeine-mediated Ca^2+^ release from ER **(E)** and KCl-mediated Ca^2+^ influx from extracellular medium **(F)**.

The new sensors, as well as R-GECO1, were next tested in cultured HEK293 cells and neurons, according to protocols that we have established for optimizing GCaMP (Tian et al., 2009; Akerboom et al., 2012a,b). The red GECIs were first tested in transfected HEK293 cells following acetylcholine (ACh)-evoked Ca^2+^ mobilization (Figures 5A,B). Subsequently, cultured rat hippocampal neurons were infected with lentivirus driving GECI expression and imaged following AP elicitation with a bath electrode (1 AP per field stimulation) (Akerboom et al., 2012a) (Figures 5C,D). In both HEK293 cells and neurons, the RCaMP and R-GECO1 sensors were expressed in both the cytoplasm and nucleus, in contrast to GCaMP, which appears to have a cryptic nuclear-exclusion sequence (Tian et al., 2009). RCaMP- and R-GECO1- expressing cells appeared healthy; thus the nuclear expression does not seem to correlate with the “cytomorbid” state seen with long-term GCaMP over-expression (Tian et al., 2009). The expression in the nucleus may give rise to a slower, weaker Ca^2+^ response (Bootman et al., 2009) than the cytoplasmic response, with the conflated epifluorescence signal thus appearing slower and less sensitive to APs.

The four RCaMP sensors tested (1d, 1e, 1f, and 1h) performed similarly well in neurons, showing observable responses following a minimum of 5 field stimuli, and reaching a maximum Δ*F*/*F* of 1.0–1.5 after 160 field stimulations. RCaMP1f and RCaMP1h were approximately twice as bright in cultured neurons as the other RCaMPs, and displayed almost identical Δ*F*/*F* and SNR. R-GECO1 was more sensitive, responding to 2 APs. SNR for R-GECO1 was also higher than for the RCaMPs, although the RCaMPs are brighter (Figure 5D). YCaMP1b showed twice the response of the RCaMPs at higher numbers of field stimuli, but required a minimum of 20 field stimuli to observe any response. However, SNR for both RCaMP1f and RCaMP1h was higher than YCaMP1b at high field stimulations, as YCaMP1b is dimmer.

The RCaMPs were also somewhat slower than R-GECO1 in the rise and decay from electrical stimulation. For the 10 AP stimulation, the time-to-half-decay *t*_1/2_(decay) and time-to-half-rise *t*_1/2_(rise) values for the GECIs were (R-GECO1: 0.78 ± 0.13/0.09 ± 0.02 s, *n* = 15; RCaMP1d: 1.61 ± 0.85/0.29 ± 0.03 s, *n* = 16; RCaMP1e: 1.57 ± 0.20/0.29 ± 0.02 s, *n* = 13; RCaMP1f: 1.80 ± 0.33/0.27 ± 0.02 s, *n* = 16; RCaMP1g: 1.69 ± 0.63/0.28 ± 0.05 s, *n* = 16; RCaMP1h: 1.83 ± 0.30/0.32 ± 0.04 s, *n* = 15; all measurements s.d.).

### Two-color sub-cellular imaging in neurons

Having established the baseline performance level of the RCaMP indicators in neurons, we sought to demonstrate the utility of RCaMP in two-color imaging, beginning by labeling two compartments of one cell. First, RCaMP1e was fused with an N-terminal cytochrome C oxidase subunit VIII tag for targeting to the mitochondrial matrix, expressed by AAV6 virus, and co-infected along with cytoplasmic GCaMP3.5 (Tian et al., 2009) (GCaMP3 without the CaM-Asn60Asp mutation; affinity ~3-fold lower than GCaMP3) into primary rat cortical neurons. Upon stimulation of Ca^2+^ release from the endoplasmic reticulum (ER) by addition of 10 mM caffeine, RCaMP fluorescence initially rose together with GCaMP fluorescence (Figure 5E), consistent with Ca^2+^ release from the ER through the mitochondrial-associated membrane (MAM) (Hayashi et al., 2009), while the bulk of Ca^2+^ released from the ER entered the cytoplasm before being buffered in mitochondria. Likewise, depolarization with 64 mM KCl demonstrated entry of extracellular Ca^2+^ into the cytoplasm and subsequently into mitochondria (Figure 5F). These experiments demonstrate the simultaneous visualization and tracking of sub-cellular Ca^2+^ fluctuations using RCaMP together with GCaMP. This paradigm may be expanded to tracking of sub-cellular Ca^2+^ mobilizations under various physiological or pathophysiological conditions in various cell types.

### Two-color imaging in mixed culture of neurons and astrocytes

**Figure 6:**
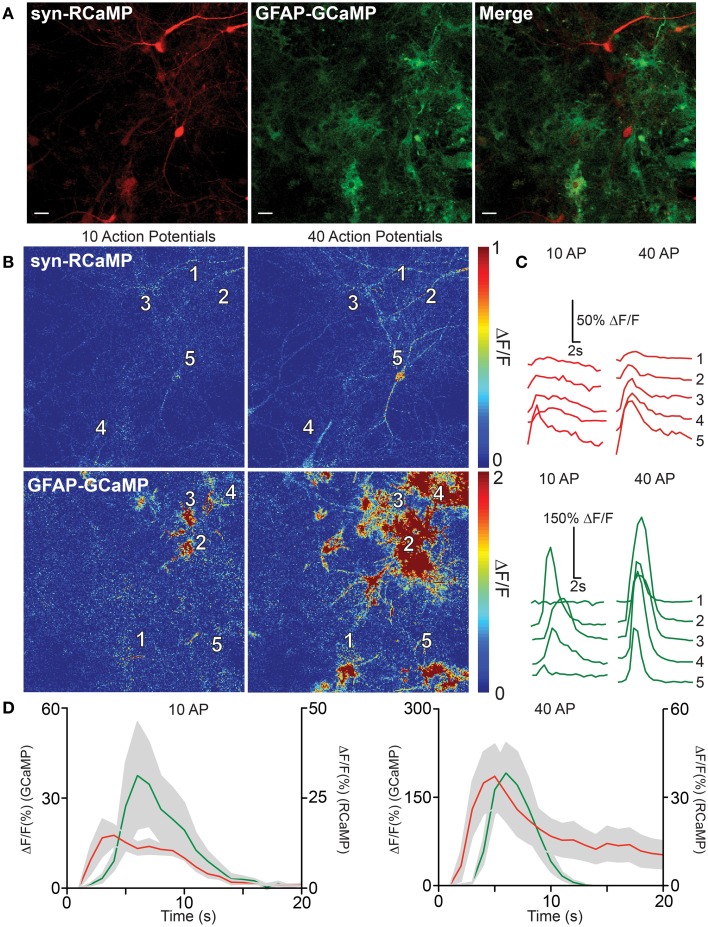
**RCaMP visualization in co-culture with GCaMP-expressing astrocytes. (A)** Expression of RCaMP1h and GCaMP5G in cultured rat hippocampal neurons and astrocytes using tissue specific promoters. Scale bars: 10 μm. **(B)** Heat maps of neuronal (top) and astrocyte (bottom) activity in response to 10 (left) and 40 (right) field stimulations. Five ROIs are specified surrounding neuron and astrocyte somata. (**C**) Single-trial neuron (top) and astrocyte (bottom) calcium activity in selected ROIs, following 10 (left) and 40 (right) evoked action potentials. **(D)** The average GECI responses of neurons (red) and astrocytes (green) following 10 (left) and 40 (right) field stimulations. Mean ± sem. (*n* = 5) shown.

Communication between astrocytes and neurons has been implicated in information processing in the brain. We therefore sought to test the utility of dual-color neuron/astrocyte imaging using RCaMP1h and GCaMP5G (Akerboom et al., 2012a) (Figure 6). RCaMP1h is 80 nm red-shifted relative to GCaMP5G; therefore signals from each population of cells are expected to be easily deconvoluted. First, RCaMP was expressed in cultured rat hippocampal neurons *via* AAV2/1-*synapsin1* virus infection; GCaMP5G was expressed in astrocytes *via* AAV2/1-*GFAP* virus (Figure 6A). The two infected cell populations were then co-cultured. One week post-infection, we observed robust labeling of neurons only with RCaMP and astrocytes only with GCaMP. Simultaneous dual-color imaging of neuronal and astrocytic responses to field stimulations was then performed. The number and magnitude of evoked calcium events in both neurons and astrocytes increased with the number of evoked APs, and most importantly, dual-color imaging revealed the spatiotemporal relationship between field stimulus-evoked neuronal and astrocytic calcium responses (Figures 6B–D). At lower number of field potential stimuli, calcium events could be detected only in astrocytes in close proximity to responding neurons (Figures 6B,C). Following larger stimuli, more neuronal activity was detected, which triggered broad astrocytic calcium events in the imaged field (Figure 6B). The peak responses of astrocytes typically lagged neuronal responses by ~1 s (Figure 6D). These results clearly show that dual-color imaging with RCaMP and GCaMP (and other indicators) holds great promise to dissect neuron-astrocyte, or other inter-cellular, communication.

### Imaging RCaMP activity in *Drosophila* larval motor neuron terminals

**Figure 7:**
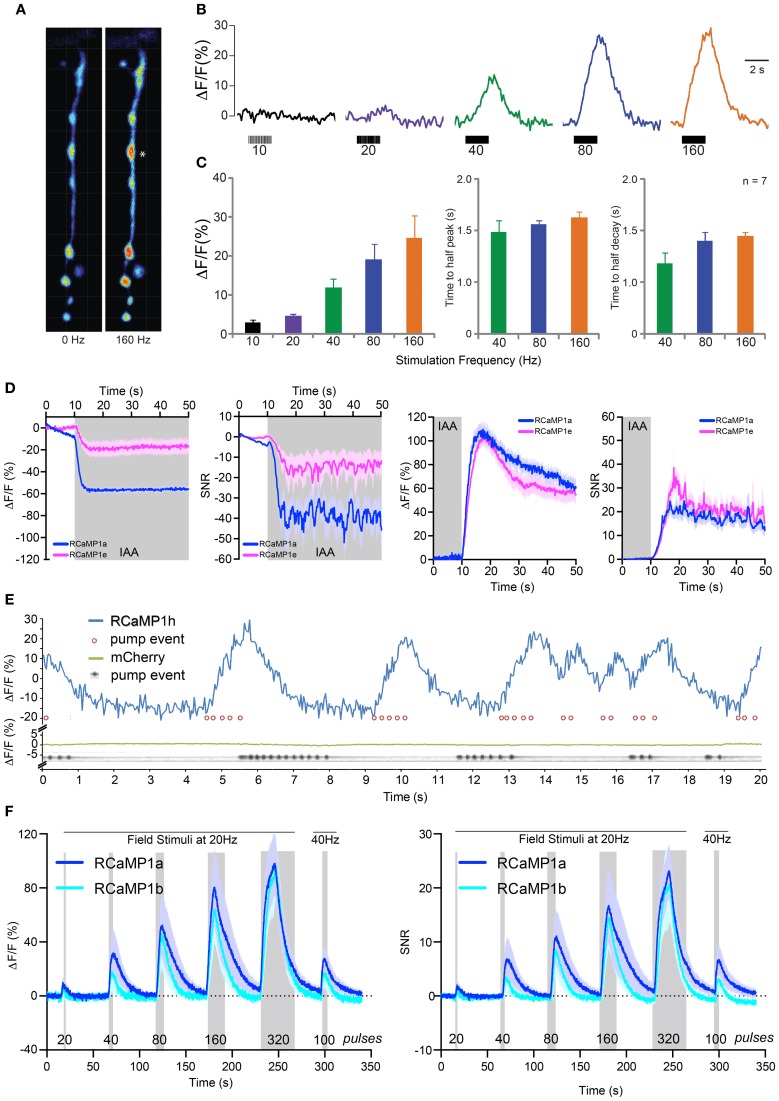
**RCaMP visualization in *Drosophila* larvae, *C. elegans*, and zebrafish. (A)** Boutons expressing RCaMP1f in *Drosophila* larval muscle 13 before (left) and at the end of (right) a 160 Hz, 2 s train of nerve stimulations. Asterisk indicates typical proximal bouton used for ROI measurements. **(B)** Raw traces from single ROI (^*^) showing percent changes in fluorescence in response to a range of stimulation frequencies. **(C)** Fluorescence change **(**mean ± s.e.m.) Δ*F*/*F* in response to increasing stimulation frequency (left), time to reach half peak amplitude (mean ± s.e.m., middle) and time to reach half decay at 40, 80, and 160 Hz (right, mean ± s.e.m.). **(D)** RCaMP1a and RCaMP1e responses to isoamyl alcohol (IAA, 10^-4^ v/v) presentation (left two panels) and removal (right two panels) in *C. elegans* AWC olfactory neurons. Both Δ*F*/*F* and SNR are shown for presentation and removal. Note that odor decreases calcium in these neurons. Traces are mean ± s.e.m. **(E)** RCaMP1h was expressed in the pharynx muscular pump and imaged for 20 s (blue trace) while the structure was pumping. Pump events were annotated based on visible movements of the terminal bulb (red circles). Ca^2+^ trace shows large increases following/concomitant with bouts of pharynx pump events. To verify that movement artifacts would not cause such signals, mCherry expressed in the pharynx muscle was analogously imaged in a separate experiment (green trace); here, pump events were detected in a kymograph showing the opening of the pharynx lumen (gray trace). **(F)** Imaging of RCaMP1a (*n* = 12) and RCaMP1b (*n* = 8) in zebrafish trigeminal neurons. Δ*F*/*F* (left) and SNR (right) in response to trains of field stimulations of increasing length (shaded region). Mean ± s.e.m. shown.

We next sought to establish RCaMP performance in *Drosophila* larvae using the GAL4-UAS system (Brand and Perrimon, 1993). We drove expression of RCaMP1f in larval motor neurons using a GAL4 driver line specific for glutamatergic neurons [*OK371*-GAL4 (Mahr and Aberle, 2006)]. Muscle 13 neuromuscular junctions (NMJs) were then imaged in dissected fillet preparations. Motor neuron terminals showed bright fluorescence; RCaMP transients in the terminals were imaged under 1-photon illumination in response to motor nerve stimulation (Figure 7A). Terminals showed little or no response to long (2 s) bursts of APs at 1–10 Hz (Figure 7B). Above 20 Hz, motor terminals showed modest peak amplitudes, peaking at ~30% Δ*F*/*F* in response to 160 Hz stimulation (Figure 7C). RCaMP responded slowly to all stimulation frequencies; times to half-peak and half-decay were ~1 s at 40, 80, and 160 Hz (rise and decay time constants could only be accurately measured at the highest stimulation frequencies).

### Imaging sensory-evoked Ca^2+^ transients in worms

We subsequently imaged RCaMP activity in the AWC neurons of *C. elegans* in response to odor addition and removal sequences, as previously described (Akerboom et al., 2012a). RCaMP1a and RCaMP1e were selected for testing. All RCaMP constructs were expressed in the AWC^on^ sensory neuron under the control of the *str-2* promoter. After 10 s of observation, odor (IAA @ 10^−4^ v/v) was added for 5 min. At the last 10 s of odor addition, imaging was commenced for another minute to observe the neuron's response to odor removal (the AWC^on^ neuron activates in response to odor removal). Interestingly, RCaMP1a outperformed RCaMP1e during IAA addition; Δ*F*/*F* and SNR of RCaMP1a were higher compared to RCaMP1e for IAA addition (Figure 7D), although RCaMP1e showed almost identical Δ*F*/*F* and SNR upon odor removal.

In addition, we demonstrated the utility of RCaMP1h in visualizing contractions of the pharyngeal muscles (strain ZX1423; *zxEx795[pmyo2::RCaMP1h; pmyo-3::eCFP]*). Worm heads were cut off (Avery et al., 1995), placed in an imaging chamber in buffer Em D50, and illuminated with a 590 nm LED (80 mW/cm^2^). As a control, worm heads of another transgenic strain (expressing mCherry in pharynx muscle) were imaged separately. RCaMP1h signal tracked pharyngeal pumping events, with large, reproducible increases up to ~40% Δ*F*/*F* (Figure 7E). In control animals, mCherry signal did not vary during the same preparation, indicating that motion artifacts play no role in the observed RCaMP signal (Figure 7E, bottom).

### *In vivo* imaging in zebrafish trigeminal neurons

We next imaged activity in trigeminal sensory neurons of 48 h post-fertilization zebrafish using RCaMP1a and RCaMP1b (Figure 7F). Trigeminal neurons are usually silent, and fire one or a few spikes in response to light touch (Douglass et al., 2008). We recorded fluorescence changes in response to trains of brief (1 ms) pulses of electrical field stimulation (Materials and Methods). Both indicators performed similarly, giving steadily increasing responses for trains of 20–320 pulses, reaching a maximum Δ*F*/*F* of about 100% (Figure 7F-left) and SNR of ~25 (Figure 7F-right).

### Integrated optogenetics/imaging with RCaMP and channelrhodopsin-2 (ChR2)

As discussed above, simultaneous light-mediated activation of neural activity (optogenetics) and fluorescent imaging of activity (either in the same or a different cell) is currently quite difficult, as the action spectra of opsins are very broad (Zhang et al., 2011), overlapping the excitation spectra of commonly used indicators such as GCaMP, Yellow Chameleon, and TN-XXL. In such regimes it is impossible to image the indicator without inadvertently driving the actuators, precluding baseline measurements. Furthermore, activators are typically driven with strong light intensities to achieve fast, reliable cell depolarization, and this can bleach indicators if they overlap. Integrated, non-overlapping imaging/optogenetics will enable qualitatively new sorts of neuroscience experiments, such as mapping projections of numerous single neurons through brain tissue, akin to an “all-optical” channelrhodopsin-assisted circuit mapping (CRACM) experiment (Petreanu et al., 2007). To facilitate such experiments, GECIs must either be red-shifted past effector action spectra, or effectors must be shifted away from GECI excitation. Significant progress has been made at red-shifting optogenetic activators (the C1V1 variant (Yizhar et al., 2011b) being the most red-shifted variant available, with maximum absorption at 550 nm); however, the action spectrum of this effector still substantially overlaps GECI excitation. Therefore, blue light activation combined with red Ca^2+^ indicators appears to be the best option for integrated optogenetics. The use of R-GECO1 and its mutants in combination with ChR2 in pyramidal neurons has been recently reported (Chang et al., 2012; Ohkura et al., 2012). However, the blue light-induced photoactivation of RGECO-1, as described above in protein and below *in vivo*, calls into question the nature of the fluorescent transients in these papers. Both papers employed mammalian neurons, which have sufficient levels of all-*trans* retinal (ATR) to support ChR2 function. In both cases, no ChR2-free controls to exclude the possibility of R-GECO1 blue-light photoactivation being the principal contributor to the observed signals were performed. Our results with R-GECO1 suggest that some or all of the observed signals previously reported on may be artifactual.

**Figure 8:**
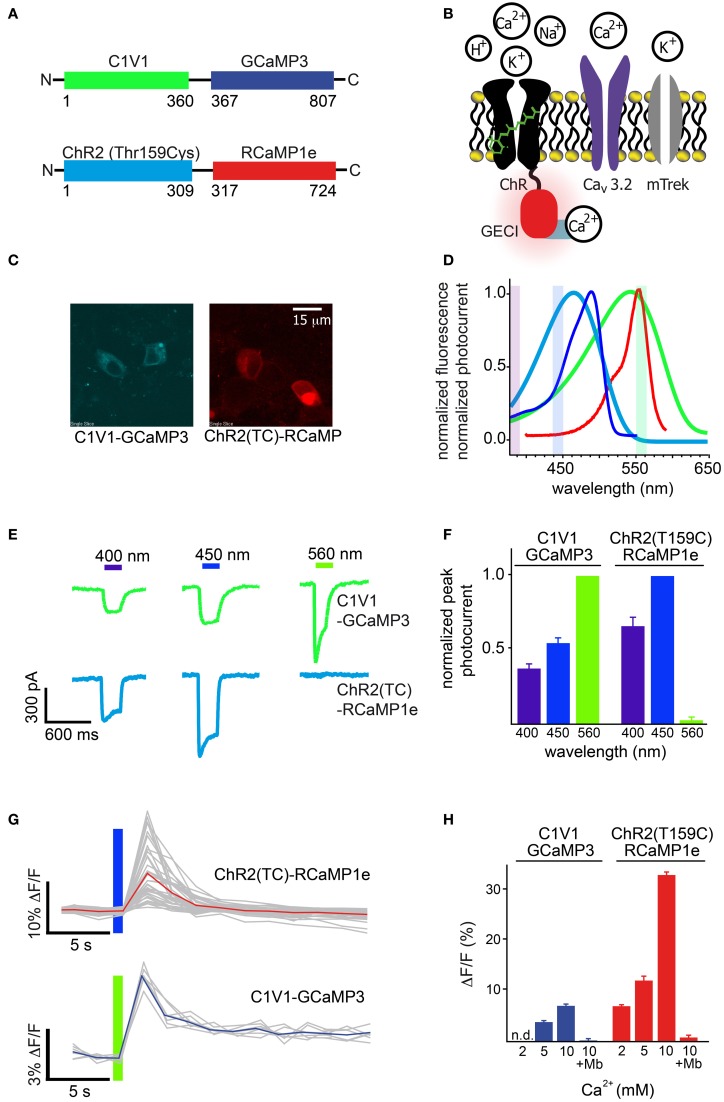
**Simultaneous optical stimulation and detection of calcium influx in HEK293 cells. (A)** Schematic of constructs, ChR2(TC) directly fused to RCaMP1e, and C1V1 directly fused to GCaMP3. Colors correspond to **(D)**. **(B)** Schematic of the channels (ChR-GECI, Ca_V_3.2, mTrek) and ions involved in the Ca^2+^ influx assay. ChR-induced membrane depolarization leads to opening of Ca_V_3.2; calcium influx is monitored via GECI fluorescence increases. The potassium channel mTrek allows hyperpolarization of HEK cells *via* extracellular [K^+^]. **(C)** Confocal fluorescence micrographs of HEK cells expressing each construct. **(D)** Normalized excitation spectra of the sensors (GCaMP3 and RCaMP1e), and action spectra of the effectors (ChR2(TC) and C1V1), colored as in **(A)**. **(E)** Typical single-trial current traces of C1V1-GCaMP3 (*green*) and ChR2(TC)-RCaMP1e *(cyan)* when illuminated with 400, 450, and 560 nm for 300 ms. **(F)** Peak photocurrent amplitudes of several (*n* = 5, 12) traces as shown in **(E)**, normalized to excitation wavelength exhibiting maximal amplitude. **(G)** Single-trial (gray) and trial-averaged (red, blue) fluorescence signal increase of each calcium sensor, after activation of Ca_V_3.2 due to membrane depolarization with C1V1 or ChR2(TC), indicated with the colored bar. **(H)** Trial-averaged peak GECI fluorescence increase in **(G)** vs. increasing extracellular Ca^2+^concentration. Addition of the Ca_V_ antagonist mibefradil (Mb) abolishes GECI responses.

First, we tested RCaMP1e together with ChR2-Thr159Cys [ChR2(TC)] (Berndt et al., 2011), as well as GCaMP3 (Tian et al., 2009) with the red-shifted channelrhodopsin C1V1 (Figure 8A). Initial experiments were performed in cultured cells, using GECIs directly fused to effector proteins (Figure 8A), in order to measure Ca^2+^ flux into the depolarized cells close to the cell membrane (Figure 8B). This arrangement creates equimolar amounts of effector and sensor, largely controlling for expression level differences in this preliminary intra-cellular assay. To measure Ca^2+^ flux we employed a stable HEK cell line expressing the leaky mTrek potassium channel and a voltage-gated calcium channel (Ca_V_3.2). In darkness, membrane voltage is controlled by extracellular potassium. Upon light-activation of ChR, cells depolarize and subsequent Ca_V_3.2 opening leads to an increase in intracellular [Ca^2+^] (Figure 8B). Expression of the two fusion constructs was comparable (Figure 8C). The excitation spectrum of GCaMP3 and the action spectrum of C1V1 show significant overlap compared to RCaMP1e and ChR2(TC) (Figure 8D). To characterize the level of cross-activation we first tested photocurrent amplitudes induced by light of 400, 450, and 560 nm, for both constructs (Figure 8E). ChR2(TC) exhibits virtually no activation at 560 nm (near the peak of RCaMP1e excitation), but can be activated with 400 and 450 nm (Figure 8F). C1V1 shows activation of more than 40% at all tested wavelengths, including GCaMP3 excitation at 450 nm (Figures 8E,F).

The separation of ChR2(TC) activation and RCaMP excitation allowed intense, simultaneous stimulation and imaging, at 450 nm (56 mW/cm^2^) and 560 nm (20 mW/cm^2^), respectively (Figure 8G-top). With 2 mM extracellular [Ca^2+^], 300 ms pulses of 450 nm light were sufficient to open calcium channels, giving rise to robust RCaMP transients (7.5 ± 0.9%, s.d. 5.43, *n* = 36), although variability between single-trial traces was relatively high (Figure 8H). Although it was also possible to image GCaMP3 fluorescence in conjunction with C1V1 activation, the spectral overlap required the excitation beam to be significantly attenuated (0.7 mW/cm^2^, 440 nm light), which produced very little fluorescence (Figure 8G-bottom). With 5 mM extracellular [Ca^2+^], 300 ms pulses of 560 nm light yielded only small increases in GCaMP3 fluorescence (3.7 ± 0.1%, s.d. 0.43, *n* = 7) (Figure 8H). Increasing extracellular [Ca^2+^] improved fluorescence signals in both preparations (Figure 8H), with the RCaMP increases reaching 35% (Δ*F*/*F*)_max_, and GCaMP3 increases reaching 6% (Δ*F*/*F*)_max_. Addition of the calcium channel blocker mibefradil abolished signals in both cases, indicating that very little Ca^2+^ is flowing directly through the opsins (Figure 8H) (Prigge et al., 2010).

**Figure 9:**
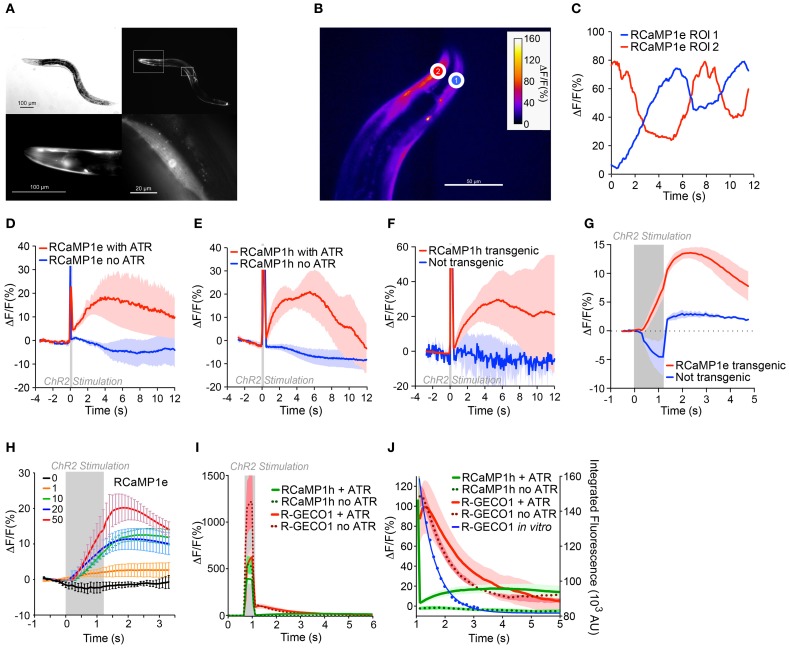
***In vivo* integrated optogenetics using ChR2 and RCaMP in *C. elegans*. (A)** Expression of RCaMP1e in body wall muscle of *C. elegans*. Differential interference contrast (DIC) image (top left) and fluorescence micrograph (top right). Lower panels show magnified regions of insets in the top right panel, i.e., head region (left) and a single body wall muscle (right). Scale bars are indicated in each panel. **(B)** Transgenic *C. elegans* expressing RCaMP1e in body wall muscle shows spontaneous muscle Ca^2+^ increases during (restrained) locomotion. A false-colored still image from the video analysis is shown, with two ROIs shown (heat map indicates fluorescence increase). **(C)** The Δ*F*/*F* in the two ROIs of **(B)** plotted vs. time. **(D)** RCaMP1e signals observed in body wall muscles directly optically excited by photostimulation of co-expressed ChR2. A 100 ms light pulse (470 nm, 50 mW/cm^2^) from an LED was presented (gray box). Animals that were raised in the presence of all-*trans* retinal (ATR) showed a robust Ca^2+^ increase that peaked ~4 s after the photostimulus, and decayed within the next 8 s (red trace, *n* = 4). Animals raised in the absence of ATR (blue trace, *n* = 2) showed no appreciable Ca^2+^ increase. Plotted are mean Δ*F*/*F* and s.e.m. **(E)** ChR2 photostimulation of cholinergic motor neurons causes a subsequent Ca^2+^ increase in body wall muscles. ChR2 was expressed in cholinergic neurons [transgene *zxIs6* (Liewald et al., 2008), using the *punc-17* promoter] and RCaMP1h in body wall muscles. A 100 ms blue light pulse (470 nm, 50 mW/cm^2^) was presented and the Ca^2+^-induced fluorescence increases were measured in single body wall muscles, in animals that were either raised in the presence (red trace, *n* = 6) or in the absence of ATR (blue trace, *n* = 3). Shown are mean Δ*F*/*F* and s.e.m. **(F)** Experiment was as in **(E)**, but comparing Ca^2+^ increases in muscle cells transgenic (red trace, *n* = 3) or non-transgenic for RCaMP1h (blue trace, *n* = 3); ATR was present in both cases. While in non-transgenic muscles only transient autofluorescence (excited by the blue light pulse) is observed, the transgenic muscles showed an increase of the fluorescent signal following the stimulus. **(G)** ChR2 expressed in cholinergic neurons was activated with 1.2 s, 50 mW/cm^2^ 470 nm light (indicated by the gray shaded region), and the Ca^2+^ increase was monitored in single muscle cells transgenic for RCaMP1e, and compared to non-transgenic muscle. Fluorescence increase in non-transgenic muscle during the blue photostimulus was subtracted from the single traces. **(H)** Experiment was as in **(G)**, with 1.2 s 470 nm light (indicated by the gray shaded region) of increasing intensities (0, 1, 10, 20, and 50 mW/cm^2^; *n* = 3, 3, 5, 6, and 4, respectively), and Ca^2+^ increases were monitored in single muscle cells transgenic for RCaMP1e and averaged across several animals. Shown are mean Δ*F*/*F* and s.e.m. **(I)** “Ca^2+^ signals” in muscles expressing R-GECO1 in animals also expressing ChR2 in cholinergic neurons. Neurons were photostimulated with a 200 ms, 50 mW/cm^2^ 470 nm light stimulus, in animals raised in the presence (thick red line, *n* = 4) or absence (red dots, *n* = 5) of ATR. Data for RCaMP1h under identical experimental conditions is shown for comparison: with (thick green line, *n* = 6) or without (green dots, *n* = 4) ATR. **(J)** Close-up of **(I)**. Blue line represents the *in vitro* data from Figure 4I (Ca^2+^-free state), showing the close correspondence between the “Ca^2+^-signals” and the blue light-induced photoactivation of R-GECO1.

Having established integrated optogenetics with RCaMP and ChR2 in cell culture, we then deployed them *in vivo* in *C. elegans*, expressing RCaMP in muscles, and ChR2 either in muscles as well, or in the upstream motor neurons. RCaMP expression was bright and restricted to muscles (Figure 9A). First, we tested the performance of RCaMP1e in visualizing spontaneous muscle contractions, in semi-restrained animals (Figure 9B). Fluorescence traces in ROIs from the dorsal and ventral sides showed clear antiphasic fluorescence increases (Figure 9C), consistent with expected muscle contractions during crawling. Fluorescence increases approached 50–60% during spontaneous muscle contraction.

Next ChR2 was co-expressed in muscles alongside RCaMP1e or RCaMP1h, to compare the responses of light-evoked contractions to spontaneously arising ones. Worms lack ATR, thus to facilitate ChR2 activation, worms were cultivated in the presence of ATR. Negative control experiments were performed in the absence of added ATR. Light pulses from a blue LED (100 ms, 470 nm, 50 mW/cm^2^) were used to activate ChR2; little photobleaching of RCaMP was seen during experiments. RCaMP was excited with a yellow-orange LED (590 nm, 79.3 mW/cm^2^), which elicits no activation of ChR2 (Figures 8D,E, 9D–H). The blue light stimulus caused a small fluorescence increase in the RCaMP channel, due to autofluorescence from the tissue (Figures 9D–F,I). To account for this, background signals were subtracted from images during the photostimulation, when prolonged stimuli were applied (Figures 9G–I). Blue light pulses evoked large, slow RCaMP transients in muscles; signals were comparable to those from naturalistic muscle contractions, although they decayed more slowly (Figure 9D). In the absence of retinal, no RCaMP signal increases were observed. Thus, the observed RCaMP signals are due exclusively to ChR2-evoked activity, and not to any potential artifacts.

Subsequently, we expressed ChR2 in cholinergic motor neurons [transgene *zxIs6*, using the *punc-17* promoter (Liewald et al., 2008)], pre-synaptic to muscle cells, and RCaMP1h in muscles. Again, we presented 100 ms blue light pulses and could detect a subsequent Ca^2+^ increase on the order of that seen from spontaneous muscle contraction and ChR2-evoked muscle depolarization (Figure 9E). The cholinergic neuron-evoked signal reached its peak after ~3 s and returned to baseline after ~12 s (Figure 9E). Again, this signal increase was only observed if retinal was added during cultivation. In muscles that were not transgenic for RCaMP, but nevertheless were innervated by ChR2-positive neurons, no fluorescence increase could be observed (Figure 9F). Similarly small amounts of autofluorescence were observed between RCaMP-positive and RCaMP-negative worms (data not shown).

We further characterized the motor neuron-induced Ca^2+^ transients in muscles expressing RCaMP1e. We presented prolonged photostimuli (1.2 s, 470 nm) to the ChR2-expressing cholinergic neurons; robust responses were seen only in RCaMP1e- positive muscles (Figure 9G). With increasing intensity of ChR2 activation, Ca^2+^ transients in muscles rose accordingly, from ~2% to ~25% (Δ*F*/*F*)_max_, for 1 and 50 mW/cm^2^ stimulus intensity, respectively (Figure 9H).

Above we showed that R-GECO1 shows dramatic reversible photoactivation by blue and green light, both in the presence and absence of Ca^2+^. We subsequently extended these results *in vivo*, imaging R-GECO1 with ChR2 expressed in cholinergic neurons. Although red fluorescence transients were observed upon blue light stimulation, identical responses were observed in worms not supplemented with ATR (Figure 9I), suggesting the signals result not from ChR2 activation of the muscles, but from photoactivation of R-GECO1. Interestingly, the half-life of fluorescence decay for R-GECO1 in *C. elegans* was similar to the half-life of photoactivation (Figures 4H,I, 9I). This illustrates that not only does R-GECO1 photoactivation produce a confounding signal to neural activity imaging, but also that the time course of the artifact is sufficiently similar to responses expected from Ca^2+^ transients following trains of APs that it may be mistakenly assigned as such (Chang et al., 2012; Ohkura et al., 2012; Wu et al., 2013).

## Discussion

We have generated a set of new chromatic GECIs based either on GCaMP or the FP mRuby. These multi-colored sensors will enable new biological experiments. We demonstrate intra-cellular two-color imaging of mitochondria/cytoplasm, and inter-cellular two-color imaging of neurons/astrocytes. Importantly, we provide a demonstration of fully integrated *in vivo* imaging/optogenetics, using functional indicators and light-gated effectors with independently addressable spectra.

New indicators are shown in the blue, cyan, yellow, and red color channels. For cyan and yellow, these are the first single-wavelength Ca^2+^ indicators in these spectral ranges. BCaMP dynamic range is comparable to B-GECO1; it is possible that the mutations in the two sensors may be additive. Systematic comparison of the RCaMP indicators with R-GECO1 reveals several advantages and drawbacks of the two scaffolds. R-GECO1 shows fast (~ms) reversible photobleaching, which partially recovers in darkness and can be pumped back to the bright state by blue or green light. In addition, R-GECO1 appears to be subject to dramatic photoactivation, rendering optogenetic implementation of this indicator problematic. These properties seem to be inherited from the mApple fluorescent protein, which shows similar photoswitching behavior (Shaner et al., 2008). No such switching behavior was observed for the sensor classes developed here. In purified protein, the RCaMP sensors were ~3 times brighter than R-GECO1 under 1-photon excitation, and twice as bright under 2-photon illumination. The two sensors showed similar, large Ca^2+^-dependent fluorescence increases in purified protein (1-photon: RCaMPs, 4-12.5×; R-GECO1, 12.5×; 2-photon: RCaMPs, 4–18×; R-GECO1, 18×), although R-GECO1 appeared to have a higher affinity for Ca^2+^. In cultured neurons, R-GECO1 showed larger and faster responses to APs. YCaMP showed large responses to long spike trains; the blue and cyan indicators likely require additional rounds of optimization before they are applicable *in vivo*.

Despite their structural similarity, the sensors manifest different mechanisms of Ca^2+^-dependent fluorescence changes. Similar to GCaMP (Nakai et al., 2001; Tallini et al., 2006; Mütze et al., 2012), the Ca^2+^-dependent fluorescence of R-GECO1 is driven largely by an increase in extinction coefficient. For GCaMP, this corresponds to a decrease in pK_a_ of the chromophore upon Ca^2+^ binding (Akerboom et al., 2009). R-GECO1 shows a small increase in quantum yield, but a nearly 10-fold increase in extinction coefficient. On the other hand, BCaMP, B-GECO1, and CyCaMP show a Ca^2+^-dependent increase in quantum yield, with little change in extinction coefficient. The responses of the RCaMP and YCaMP variants are roughly equally divided between the two.

Crystal structures of mRuby at low and high pH revealed a *cis*-*trans* chromophore isomerization in acidic environments. The high-resolution crystal structure of RCaMP1a shows that the relative conformation of the FP and CaM domains is significantly rotated relative to the GCaMP structures (Wang et al., 2008; Akerboom et al., 2009), as well as the R-GECO1 structure reported here. The adventitious mRuby-CaM interface in RCaMP shows that different CaM residues are recruited than in GCaMP, and further rationalizes many of the mutations to mRuby, CaM and the two inter-domain linkers that gave rise to and improved RCaMP responses. Delineation of this interface should facilitate additional rounds of targeted mutagenesis to improve function, as was demonstrated for GCaMP (Tian et al., 2009; Akerboom et al., 2012a). A large number of the R-GECO1 mutations found during initial optimization are present in the FP-CaM interface as well.

Interestingly, in the crystal structure of RCaMP we observed an imine hydrolysis between Phe213 and Met214, resulting in a cleavage of the polypeptide backbone at the chromophore, and SDS-PAGE showed corresponding fragmentation of a significant portion of the purified RCaMP samples. For R-GECO1, no backbone cleavage could be detected in the crystal structure, however, during SDS-PAGE, a similar fragmentation as for RCaMP is visible. Given the observation of the backbone cleavage both for SDS-PAGE and crystal structures, it is quite possible that the red GECIs exist as split proteins *in situ*. It is unclear what effect this backbone cleavage has on the sensor properties of RCaMP and R-GECO1, as we could not separate the cleaved and uncleaved species in order to study them independently. The significant red-shifting of RCaMP relative to mRuby may result from this backbone cleavage, perhaps relieving strain in the mRuby chromophore. The RCaMP indicators are also brighter under 2-photon excitation than the parent FP mRuby, in part due to the higher QY and photostability of RCaMP.

The crystal structure of R-GECO1 reveals that the relative orientation of the mApple and CaM domains is significantly shifted relative to that in GCaMP, and thus relative to the homology model used to first rationalize the R-GECO1 mutations (Zhao et al., 2011a). The selection of proline in the first linker of B-GECO1, GEM-GECO1, and R-GECO1 is consistent with our results for GCaMP (Akerboom et al., 2012a) and bacterial periplasmic binding protein-based sensors for maltose (Marvin et al., 2011), organophosphorous (Alicea et al., 2011), and glutamate (Marvin et al., 2013). Mutations selected in the creation of GCaMP, RCaMP, and R-GECO1 are concentrated in the interface between CaM and the cpFP domain. The proper packing of such proto-interfaces, and presumably the resulting regulation of the conformational change between ligand-free and ligand-bound forms, has thus emerged as a critical factor in all cpFP-based sensors developed to date. Intriguingly, the Lys47Val and Thr49Val mutations of “R-CaMP1.07” (Ohkura et al., 2012) (a double mutant of R-GECO1) are at the bottom of the cp-mApple barrel, and the mechanism of their apparent improvement remains unknown.

We have illustrated the design and optimization of several new chromatic classes of calcium indicators, and applied RCaMP to *in vivo* imaging in worms, zebrafish, and the *Drosophila* larval NMJ. Photobleaching under 2-photon excitation currently limits deep *in vivo* imaging for both RCaMP and R-GECO1. Improved versions of these indicators, with greater photostability and response to APs (and no confounding photoactivation), could allow deep multi-photon functional imaging, with lower background and phototoxicity. Additionally, it may be advantageous to target the new GECIs for nuclear exclusion (Krebs et al., 2012); cytoplasmic Ca^2+^ responses to APs are stronger and faster than nuclear signals (Bootman et al., 2009), and conflating the two [RCaMP and R-GECO1 do not appear to have cryptic nuclear exclusion sequences as does GCaMP (Tian et al., 2009)] may artificially depress and lengthen transients.

We demonstrate 2-color imaging using RCaMP together with the green sensor GCaMP, both between cell compartments within cells, and between different cell types. We have recently used RCaMP, in conjunction with a green sensor of extracellular glutamate (Marvin et al., 2013), to simultaneously image synaptic input into, and output from, neurons in *C. elegans* (Marvin et al., 2013). Importantly, we show robust integration of RCaMP with optogenetics, with orthogonal activation and functional imaging, both in cultured cells and in partially restrained *C. elegans*. We have recently used this technique to demonstrate functional, genetically specified synaptic connections in living worms by all-optical methods (Husson et al., 2012). Simultaneous, independent addressing of activation and imaging will enable “closed loop” optogenetics experiments, wherein functional imaging data is used dynamically to update activation. We show that R-GECO1 is not suited for use in optogenetic experiments due to its blue light-dependent photoswitching behavior. It might be possible that using a reduced intensity level of blue stimulation together with the proper ChR2- or ATR-null controls, these photoswitching effects with R-GECO1 could be minimized, or accounted for. Showing no signs of photoactivation, though, the RCaMP sensors are the best-suited indicators for simultaneous, and eventually “closed-loop,” optogenetics and functional imaging.

## Materials and methods

### Initial RCaMP construction

For the red protein in RCaMP we selected mRuby (Kredel et al., 2009). DNA encoding mRuby was generated by assembly PCR (Stemmer et al., 1995), and cloned in pRSETa (Invitrogen, USA). We decided to test two different circular permutation sites in mRuby; (1) between Pro142 and Thr143 in beta strand seven [homologous to cpEGFP in GCaMP (Nakai et al., 2001)], and (2) between His196 and Arg197 in beta strand 10 [in close proximity to a potential planar *trans* conformation of the chromophore in mRuby, as described for its parental protein eqFP611 (Petersen et al., 2003)]. M13pep and CaM DNA, including linkers, were identical to their counterparts present in GCaMP3 (Tian et al., 2009). We also swapped the M13pep and CaM domains for each circular permutation site. Assembly of the four resulting RCaMP constructs was performed as follows; the four different parts (the N-terminal (from Met1 to Pro142 and from Met1 to His196) and C-terminal part (N143 to G227 and Arg197 to G227) of mRuby, the DNA encoding the M13pep including (M1 to E60) and DNA encoding CaM (T302 to K450) (mRuby and GCaMP3 numbering, respectively) were PCR amplified from GCaMP3 and mRuby DNA. Seven codon overlaps were encoded in the primers. PCR products were purified by gel extraction, pooled in equimolar amounts for the corresponding constructs, and assembled in a PCR assembly reaction. One to five microliter of this reaction was then used as a template in a following PCR reaction using primers annealing to the N-terminus and C-terminus of the final PCR product. Resulting DNA was gel purified, digested with NdeI and HindIII and ligated into pre-digested pRSETa and sequenced for verification.

### Directed evolution and rational optimization of RCaMP

#### M13pep-cpmRuby linker screen

Only BL21(DE3) colonies containing the RCaMP construct with the circular permutation site between Pro142 and Thr143 and the M13pep on the N-terminus and CaM attached to the C-terminus displayed very dim red fluorescence after 120 h incubation at 4°C. To improve maturation of RCaMP, we chose to first randomize the M13-cpmRuby linker Leu-Glu to either three amino acids (XXX) or two amino acids (XX) by Kunkel mutagenesis (Kunkel et al., 1991) and screen for faster maturing variants. Mutagenesis was performed as described previously (Akerboom et al., 2009, 2012b). Kunkel reactions were transformed into *E. coli* XL1-Blue (Stratagene), successful transformants were scraped off plates, pooled together, and the linker libraries in pRSETa were isolated using the Qiagen Miniprep kit (Qiagen, Germany). One microliter of each library was subsequently transformed to BL21(DE3) for high-level expression, plated on 244 mm × 244 mm LB-agar plates, resulting in two libraries of approximately 10,000 variants each. Plates with colonies were stored at 4°C, and were checked for fluorescence after 12, 24, 36, 48, and 60 h using an Olympus MVX10 microscope with proper lamps and filter sets. A total of 384 clones were selected for analysis by picking colonies that displayed the strongest red fluorescence at each time point. Candidates were grown for two days at 30°C in ZYM-5052 auto-induction media (Studier, 2005) containing ampicillin in 96-well deep well blocks. Five microliter of each culture was used to inoculate 2YT media for plasmid purification and sequencing.

Fluorescence intensity and signal-change measurements of variant libraries were performed in bacterial lysate as follows: BL21(DE3) cell pellets were resuspended in 400 μl lysis buffer (20 mM TRIS.HCl, 1 mM MgCl_2_, 1 mg/ml lysozyme, 1.5 Kunitz units DNase-I/ml) and incubated at 30°C while shaking (120 rpm) for 2 h. Lysates were subsequently clarified by centrifugation (20 min, 4000× *g* at 4°C). Ninety-five microliter of each sample was transferred to a UVStar 96-well plate (Greiner, Germany) and fluorescence was measured in a Tecan Safire^2^ (Tecan Group Ltd., Switzerland) spectrophotometer at 585 nm excitation—610 nm emission wavelengths before and after subsequent additions of 2 μl 0.1 M EGTA and 5 μl 0.1 M CaCl_2_. Four faster maturing variants with a calcium-dependent fluorescence increase greater than 100% in *E. coli* lysate were selected and the corresponding plasmids were sequenced. The four candidates contained the following M13-cpmRuby linkers: Cys-Ile (RCaMP-CI), Arg-Ile (RCaMP-RI), Pro-Arg-Ile (RCaMP-PRI), and Ala-Ile (RCaMP-AI). For each of the different linkers multiple candidates were selected with different codons for the isoleucine present in all the variants selected, ruling out enrichment of specific clones during the mutagenesis reaction for this amino acid and indicating the importance of an isoleucine at this linker position.

To analyze this set of four RCaMP variants as purified proteins, we re-transformed the plasmids into *E. coli* BL21(DE3), and inoculated 500 ml ZYM-5052 (Studier, 2005) containing ampicillin. The cultures were allowed to grow at 200 rpm shaking at 37°C until visibly turbid, after which the temperature was lowered to 30°C. Cultures were incubated at 30°C for 36 h while shaking at 200 rpm. Protein was purified via nickel affinity purification as described previously (Akerboom et al., 2009). Comparing fluorescence of purified proteins under calcium-free and calcium-loaded conditions gave Δ*F*/*F*_0_ values of 2.2 ± 0.1 for RCaMP-CI, 2.3 ± 0.05 for RCaMP-RI, 1.8 ± 0.2 for RCaMP-PRI, and 2.7 ± 0.1 for RCaMP-AI. Due to the potential reactivity of the cysteine in the M13 linker of RCaMP-CI we decided to continue with RCaMP-RI, RCaMP-PRI, and RCaMP-AI.

#### Random mutagenesis

To further optimize speed of maturation and brightness, we performed random mutagenesis on RCaMP-AI, RCaMP-RI, and RCaMP-PRI using error-prone PCR. The Genemorph II kit was used according to the manufacturer's instructions (Stratagene, USA). Primers were designed to anneal directly outside of RCaMP in the pRSETa construct. Template concentrations were set to generate around 3 mutations per kb. PCR mutagenesis products were gel-purified, digested with NdeI and HindIII, ligated into pRSETa and subsequently transformed to *E. coli* XL1-Blue. Transformants were scraped off agar plates, pooled, and plasmids were isolated with the QIAGEN miniprep kit (Qiagen). Five microliter of purified plasmid DNA was subsequently transformed to *E. coli* BL21(DE3). Colonies were allowed to develop fluorescence for 24 h at 4°C before being checked for red fluorescence under a wide-field stereomicroscope equipped with a red filter set. Four hundred variants that developed visible red fluorescence after 24 h were selected and grown for two days at 30°C in ZYM-5052 media (Studier, 2005) containing ampicillin in 96-well deep well blocks. Five microliter of each well was subsequently used to inoculate 2YT media for plasmid purification and sequencing. The ZYM-5052 cultures were pelleted and stored at −20°C. Bacterial lysate preparation and fluorescence measurements were performed as described for the M13pep-cpmRuby screen. From this random mutagenesis screen, four variants with improved fluorescence maturation characteristics stood out: RCaMP-PRI-1 (G21D, T148I, S151R, T364M, N391S, N431Y), RCaMP-PRI-2 (P224Q, G109R, E325A), RCaMP-RI-1 (Y25Q, T219I, S311P), RCaMP-AI-1 (T148A, A210V, D372G).

#### Directed mutagenesis

Mutagenic sites resulting from the random mutagenesis screen, as well as T60, D61, H111, A112, V113, and H115, chosen from inspection of the eqFP611 (Petersen et al., 2003) and mRuby crystal structures, were selected for saturation mutagenesis. We chose to use RCaMP-AI as a template, because it had the highest Δ*F*/*F*_0_ of the four M13-cpmRuby linker variants. The site-specific libraries were created by Kunkel mutagenesis using primers containing NNS at the codon of interest as described for the M13pep-cpmRuby, and were screened as described for the PCR mutagenesis mutants, except that colonies were not screened for red fluorescence but instead were picked randomly with a QPix2^*XT*^ colony picker (Genetix, UK).

Four mutations that improved RCaMP brightness, maturation and Δ*F*/*F*_0_ significantly (G109A, A112Y, T364I, and D372Y) were combined to produce RCaMP1c. One additional spontaneous mutation, A270V, which lowered affinity and ΔF/F *in vitro*, but sped up the maturation dramatically, was also incorporated and was named RCaMP1d.

To further enhance RCaMP1d, we randomized positions K136, T294, and the cpmRuby-CaM linker R295-D296, tested the following directed mutations: L56Q, I60L, H77I, H77E, V140F, V140L, R384G, and I424S. These mutations were located at the interface between domains in the crystal structure, had close proximity to the domain linkers, or were predicted to influence calcium binding. We screened combinatorial libraries of these mutants as described for the M13pep-cpmRuby linker variants, using the QPix2^XT^ colony picker (Genetix, UK) to randomly select variants. Mutations which improved Δ*F*/*F*_0_ or affinity were the cpmRuby-CaM linker R295D-D296S, R384G, and I424S. Combinations of these mutations resulted in two final RCaMP variants; RCaMP1f and RCaMP1h (Table 1, Figure 2A).

### Construction of YCaMP, CyCaMP, and BCaMP

YCaMP, CyCaMP, and BCaMP were constructed by Quikchange mutagenesis (Stratagene, USA) using GCaMP3 (Tian et al., 2009) in pRSETa as template. Reactions were performed according to the manufacturer's instructions and constructs were sequenced for verification. We used combinations of published mutations to generate the different excitation and emission characteristics. For YCaMP, we used V115Y and K118V to shift the excitation/emission to yellow. For cyan, we used Y223W and Y223W in combination with S229A to generate CyCaMP1a and CyCaMP1b, respectively. For BCaMP, we initially mutated Thr222Ser and Tyr223His. This resulted in a very dim non-responsive BCaMP1a, so we screened variants of both the M13pep-cpEBFP and cpEBFP-CaM linker as described above. We found two linker variants (LeuGlu to MetPro for M13pep-cpEBFP linker and ThrArg to PhePro for cpEBFP-CaM linker) with improved brightness and Δ*F*/*F*_0_.

### Protein expression and purification

Proteins were expressed and purified as described previously (Akerboom et al., 2009), with the following adaptations: proteins were expressed at 30°C for 36 h for RCaMP and at 25°C for the other color variants.

### Size exclusion chromatography

To examine the oligomeric state of RCaMP, an aliquot of purified protein in 20 mM Tris, pH 8.0, 100 mM NaCl, 2 mM CaCl_2_ was injected onto a Superdex 200 SEC column with 20 mM Tris, pH 8.0, 100 mM NaCl, 2 mM CaCl_2_ as the running buffer.

### Crystal structure determination

mRuby, RCaMP, and R-GECO1 proteins were expressed in *E. coli* BL21(DE3) from the pRSETa plasmid (Invitrogen). Proteins were purified by immobilized metal ion affinity and SEC. The N-terminal tag encoded by the pRSET vector was cleaved from mRuby proteins using enterokinase prior to SEC. Proteins were concentrated to ~10 mg/mL for crystallization using centrifugal concentrators (Millipore) with a 10 kDa molecular weight cut-off (MWCO). Crystallization using commercially available sparse matrix screens (Hampton Research) was done by mixing 1.2 μL of protein solution with 1.2 μL of precipitant in 96-well plates. X-ray diffraction data were collected at 100 K at the LRL-CAT beamline (31-ID) of the Advanced Photon Source. X-ray diffraction data were processed using Mosflm (Leslie, 1992; Battye et al., 2011)/Scala (Winn et al., 2011) (Table 2). Structures were solved by molecular replacement using Phaser (McCoy et al., 2007) with previously published structures of EqFP611 (PDB 3E5W) (Petersen et al., 2003), mCherry (PDB 2H5Q) (Shu et al., 2006) and CaM-M13 (from GCaMP, PDB 3EK7) (Akerboom et al., 2009). Iterative cycles of model building in Coot (Emsley et al., 2010) and refinement in Refmac/CCP4 (Winn et al., 2011) led to the final models described in Table 2.

### Spectrophotometric analysis of purified protein

Fluorescence spectra were obtained as described previously (Akerboom et al., 2009). In short, pure proteins were dialyzed into 20 mM TRIS.HCl, pH 8.0, 100 mM NaCl and diluted 10-fold into zero-free calcium buffer (Invitrogen) for calcium-free spectra, and in 39 μM free-calcium buffer (Invitrogen) for calcium-loaded spectra. For absorbance spectra, undiluted samples were taken and EGTA or CaCl_2_ was added to a final concentration of 10 mM. Affinity measurements were done in calcium standards prepared by mixing different amounts of 39 μM free calcium buffer and zero calcium free buffer together according to http://maxchelator.stanford.edu (Patton et al., 2004) and the manual provided with the calcium kit. Measurements were taken in a Tecan Safire^2^ (Tecan Group Ltd., Switzerland), and data was analyzed using the GraphPad Prism software package (http://www.graphpad.com/prism/Prism.htm).

### Stopped-flow kinetic analysis

The kinetics of calcium release by the indicators was determined from a single exponential fit to the fluorescence decay following rapid mixing of 1 μM protein samples in 10 μM calcium with a solution of 10 mM EGTA at room temperature, both buffered with 50 mM MOPS, 100 mM KCl at pH 7.2, using a stopped-flow device (Applied Photophysics) coupled to a fluorometer (Varian).

### pH titrations

The pKa of both calcium-bound and calcium-free fluorescent indicators was determined by measuring the fluorescence at different pH values. Purified protein was diluted 20-fold into buffer A (20 mM Glycine, 20 mM Citrate, 20 mM TRIS.HCl) at different pH values, containing 2mM CaCl_2_ for calcium-loaded spectra and 2 mM BAPTA for calcium-free spectra.

### SDS-PAGE analysis

For SDS-PAGE, small (15 μL) protein samples were mixed with 5 μL LDS Sample buffer (Life Technologies, USA) supplemented with 2-Mercaptoethanol, incubated at 100°C for 5 min, cooled on ice, and loaded onto pre-cast NuPAGE Bis-Tris gels (Invitrogen), and run at 50 V in MES buffer provided, until satisfactory migration of protein bands was reached. Protein mass was determined by running 5–10 μL SeeBlue Plus2 protein marker (Life Technologies, USA) in parallel. Gels were stained with staining buffer [50% methanol, 10% glacial acid, 40% distilled water, 1 g/L coomassie brilliant blue R-250 (FisherBioscience)], and destained with destaining buffer (similar to staining buffer, with coomassie brilliant blue omitted). Gels were imaged using an AlphaImager HP (ProteinSimple, USA), and sizes were calculated using the AlphaEaseFC software package (Genetic Technologies, Inc., USA).

### Photophysical measurements

Photophysical measurements were taken on purified protein in buffer (30 mM MOPS, 100 mM KCl) in the absence (10 mM EGTA) or presence (10 mM Ca-EGTA) of free Ca^2+^ (calcium calibration buffer kits, Invitrogen). All solutions had a measured pH of 7.28 ± 0.08. Absorption measurements were performed on a UV/VIS spectrophotometer (Lambda 35, Perkin Elmer) with 2-nm slit settings. Emission spectra were taken on a fluorescence spectrometer (LS55, Perkin Elmer) with 5 nm slit settings, using a quartz microcuvet to obtain excitation spectra in the UV in addition to the visible range. Quantum yield measurements were performed with an integrating-sphere spectrometer (Quantaurus, Hamamatsu), using protein solutions with an absorbance of about 0.1 OD at 570 nm. Two-photon spectroscopy measurements were performed on an inverted microscope (IX-81, Olympus) outfitted with a 60× 1.2 NA objective, using an 80 MHz Ti:Sapphire laser (Chameleon, Coherent) spanning 700–1080 nm, as described previously (Akerboom et al., 2012a; Mütze et al., 2012). An extended wavelength range (1000–1400 nm) was accessed by the addition of an OPO (Chameleon OPO, Coherent). Fluorescence collected by the objective, after passing through shortpass and bandpass filters (720SP, 625/90, Semrock), was detected using a fiber-coupled avalanche photodiode (APD) (SPCM_AQRH-14, Perkin Elmer), which operates in single-photon counting mode. Output pulses from the APD were fed to an autocorrelator (Flex03LQ, Correlator.com). This system was run under computer control with automated data acquisition, and allowed us to determine the two-photon-excited fluorescence excitation spectra. To perform time-resolved fluorescence lifetime measurements, we employed a fast-timing APD (PDF-CCTB, Micro Photon Devices) fed into a single-photon counting board (TimeHarp200, PicoQuant). We used two-photon-excited fluorescence correlation spectroscopy (FCS) to quantify chromophore concentration and two-photon specific brightness using EGFP at known concentration as a reference to calibrate the excitation volume. All FCS autocorrelation fits were performed using a custom fitting program in MatLab (Vijay Iyer, unpublished). We also used FCS to determine the fluorescence rate per molecule, or specific brightness, given by <*F*>/*N* = <*F*>*G*(0), as the laser power is varied. The peak brightness, the maximum value of the specific brightness, provides a metric to compare the photostability under two-photon illumination (Mütze et al., 2012). One-photon photobleaching measurements were made in an upright microscope (Imager.Z2, Zeiss), using a 20× 0.85NA objective, with illumination from either a Hg-lamp (excitation filter 550/25, Semrock), or a 561-nm laser (Sapphire 561 LP, Coherent). The intensity at the sample was 2.7 W/cm^2^ for lamp illumination, and 6.0 W/cm^2^ for 561-nm laser illumination. Samples consisted of proteins in aqueous buffer droplets (50 mM MOPS, 100 mM KCl, 1 mM CaCl_2_, 0.1% BSA, pH 7.3) suspended in a nonpolar solvent (1-octanol) between two coverslips, following the method of Kremers and Piston (2010). Fluorescence from individual droplets contained within the area of uniform illumination was collected by the objective, passed through a bandpass filter (605/70, Semrock), and sent to either a CCD camera (CoolSnapEZ, Roper) or an APD to record the emission with high temporal resolution. In order to investigate photoswitching in the protein samples, additional illumination by a 405 laser (Sapphire 405 LP, Coherent) was combined along the path of the 561-nm laser. All light sources were outfitted with shutters, and exposure sequences were set by a custom software program.

### Transient absorption and emission of R-GECO1 following 488-nm excitation

Absorption and emission properties of R-GECO1 and RCaMP1h were measured using a sub-microcuvet (16.40F-Q-10/Z15, Starna Cells) of 0.036 cm^2^ area and 1 cm path length, in a setup providing 488-nm laser light delivered to the cuvet under shutter control, and a counter-propagating probe beam of lamp-light transiting through the same area of the cuvet. The transmitted probe light was sent to a fiber-coupled CCD-based spectrometer (QE65000, Ocean Optics), which captured and stored full absorption spectra of 100-ms duration at a frame rate of 10 s^−1^. Optical density was calculated from -Log[S(1)/S_o_(1)], where S_o_(1) is the lamp-light signal recorded at each wavelength of the spectrometer in the absence of R-GECO1 and S(1) the signal measured with R-GECO1 present. Due to baseline drifting for this setup, we found it useful over the course of long experiments to determine just the transient absorption, ΔOD, from −Log[S_post-488_(1, t)/S_pre-488_(1)].

Transient fluorescence was recorded in this setup from side-window measurements of the cuvet. Emission light was focused onto a fiber-coupled florescence spectrometer (Pixis256E CCD + SB-2358 Acton spectrograph, Princeton Instruments), capable of recording 100 ms per spectra at a frame rate of 7.9 s^−1^. The transient fluorescence was excited by weak 561 nm light (0.025 W/cm^2^). Measurements at 10-fold higher intensity yielded equivalent results, indicating the weak 561 nm light was not perturbing the photophysics.

Excitation laser light overfilled the 0.036 mm^2^ open area of the cuvet, and incident intensities for 488, 405, and 561 nm were determined from measurements of laser power transmitted through the cuvet containing buffer only, divided by the open area of the cuvet (0.036 cm^2^). Laser exposure time was controlled with a shutter (Uniblitz) under software control. The spectrometers were operated in free-running data-acquisition mode, i.e., not synchronized to the laser shutter. However, we could determine accurately the timing from the end of the 488-pulse to the beginning of the first and subsequent data frames from laser fiducials appearing in each frame.

Samples of R-GECO-1 or RCaMP1h were diluted in the microcuvet containing buffer (MOPS/KCl/10mM-EGTA, pH 7.25), and data taken for this “no-calcium” case. Following these measurements, CaCl_2_ was added to provide 10 mM final concentration in the buffer, the sample allowed to equilibrate, and measurements performed for the “+ Ca” case. Absorption experiments were performed for 3 concentrations (5, 10, 20 μM) and fluorescence experiments performed at 10 μM and 3 μM, in order to assess possible concentration effects (none observed).

### HEK293 cell acetylcholine Ca^2+^ mobilization assay

RCaMPs were cloned into pCAGGS by PCR amplification, subsequent digestion with AgeII and NotI and ligation into predigested pCAGGS (Niwa et al., 1991). Constructs were sequenced for verification. Transfections were done in low passage number HEK293 cells using the Amaxa 96 nucleofector kit (Lonza Group Ltd., Switzerland). Cells were then incubated for 48 h in DMEM.HG (Corning, USA) supplemented with 10% Fetal Bovine Serum (FBS) at 37°C, 10% CO_2_. Media was renewed after 24 h. After 48 h, growth media was aspirated and 100 μl 1× TBS buffer supplemented with 2 mM CaCl_2_ was gently pipetted on top of the cells. Imaging was performed immediately afterwards in an FDSS plate reader (Hamamatsu, Japan). Dilutions of acetylcholine in 1× TBS supplemented with 2mM CaCl_2_ were added 10 s after read initiation. Data was analyzed using the GraphPad Prism software package (http://www.graphpad.com/prism/Prism.htm).

### Hippocampal and cortical neuronal culture imaging

Procedures were as described previously (Akerboom et al., 2012a). Briefly, dissociated hippocampal or cortical neurons obtained from P0 rat pups were plated in 24-well plates and transduced with GECI variants expressed from the human *synapsin-1* promoter using lentivirus or adeno-associated virus (AAV). Fluorescent signals were imaged concomitant with trains of APs at 30 Hz, induced by field electrode stimulation. Imaging data were analyzed using a customized algorithm in MATLAB (MathWorks).

### Channelrhodopsin—HEK293 cell measurements

All fluorescence experiments were performed on an IX70 microscope (Olympus, Tokyo, Japan) with a 40× and 1.35 N.A. oil objective (Fluar Zeiss, Germany). For dual-wavelength excitation of ChR and RCaMP1e or GCaMP3, two independent light sources were applied. For RCaMP1e and GCaMP3 excitation we used a Polychrome V unit (TILL Photonics, Planegg, Germany) set to 560 nm or 440 nm (FWHM 7 nm), respectively. ChRs were activated with a 75W-Xenon lamp (Leistungselektronik Jena, Jena, Germany) bandpass filtered either with 450 nm (FWHM 15 nm) or 560 nm (FWHM 15 nm) (AHF Analysentechnik, Tübingen, Germany). Both light sources were controlled by the Tillvision Software (TILL Photonics, Planegg, Germany). The xenon lamp was modulated via a fast shutter (Uniblitz, Rochester, NY). Fluorescence was selected via a dichroic mirror (FF01-468/553-25, Semrock, Rochester, NY) and projected onto a CCD camera (Imago, TILL Photonics, Planegg, Germany). Fluorescence was sampled every 3 s at an exposure time of 500 ms and a resolution of 320 × 20 pixels (2 × 2 binning). Δ*F*/*F* was calculated by subtracting the baseline fluorescence level (average of 20 data points before ChR activation) and subtracted from the actual fluorescence response and normalized to *F*_0_. All fluorescence data was background corrected.

Electrophysiology was performed as previously described (Prigge et al., 2010). Briefly, patch pipettes were pulled from micro-haematocrit-tubes (Hecht-Assistant, Sondheim, Germany). Pipettes were then filled with (in mM): 110 NaCl, 10 EGTA, 2 MgCl_2_, 2 CaCl_2_, 2 KCl, and 10 HEPES buffer (pH 7.5). External solution contained (in mM): 140 NaCl, 2 KCl, 2 MgCl_2_, 1, 2, 5, or 10 CaCl_2_ and 10 HEPES (pH 7.2). pH and osmolarity were adjusted with NMG/HCl to 7.2 and Glucose to 280 or 320 mOsm, respectively. Voltage clamp measurements were performed with a HEKA EPC 7 amplifier (Heka Electronics, Darmstadt, Germany) and visualized with pClamp Software (Molecular Devices, Foster City, CA).

A HEK293 cell line constitutively expressing Ca_V_3.2 and mTrek (Yao et al., 2006) was transfected one day after seeding using Fugene HD (Roche, Indianapolis, IN). Fluorescence or electrophysiology was performed one day after transfection. ATR was added into cell growth media to a final concentration of 1 μM.

C1V1 or ChR2(TC) were fused C-terminally to GCaMP3 or RCaMP1e by overlap-extention PCR. The product was cloned into pECFP-N1 (Clontech, Mountainview, CA) via NheI and XbaI digestion and subsequent ligation.

### Imaging GECIs in *C. elegans* with channelrhodopsin photostimulation

For Ca^2+^ imaging of body wall muscles, worms were fully or partially immobilized by gluing them with surgical glue (Octyl Cyanoacrylate) onto 2% agar pads in M9 buffer. RCaMP1e and RCaMP1h fluorescence was measured on an inverted fluorescence microscope (Zeiss, Axio Observer) equipped with two high power light-emitting diodes (LEDs; 590 and 470 nm, KSL70 Rapp Optoelektronik), coupled with a beam splitter that allows simultaneous illumination at two wavelengths, for excitation of RCaMP, as well as for photostimulation of ChR2. The filter set for imaging consisted of a double band pass excitation filter 479 and 585 nm combined with a 605 nm beam splitter and a 647/57 band pass filter for emission (AHF Analysentechnik: F74-423, F38-605, F37-647). Illumination protocols were run using the Lambda SC Smart Shutter controller (Sutter Instruments). Videos were obtained with a CCD camera (Photometrics Coolsnap HQ^2^, Roper Scientific). Calcium traces were taken by a 40× oil immersion objective (Zeiss EC Plan Neofluar 40× 1.3 NA) and analyzed using ImageJ (http://rsbweb.nih.gov/ij/index.html), generating ROIs for single or few muscle cells that did not show major movement. As a control, muscles in animals raised in the absence of ATR were analyzed, or non-transgenic muscles next to the measured transgenic muscle (animals contained extra-chromosomal arrays of RCaMP1e, and expression was thus subject to minor mosaicism). Data were calculated to obtain Δ*F*/*F*. For some experiments involving prolonged photostimulation, the fluorescence increase due to the autofluorescence was deduced by taking the fluorescence value of the first video frame of the blue stimulus, and subtracting this value from each measured fluorescence value during the photostimulus. To obtain normal fluorescence images, the same microscope was used with a HBO 100 lamp and a filter set for red fluorescent probes, e.g., Cy3. Some animals were only partially immobilized by gluing, to allow motility, and the ROIs were then traced manually for analysis, using non-ambiguous anatomical landmark features that could be followed even under fluorescence imaging. For Ca^2+^ imaging of the pharynx, heads were cut off and placed in an imaging chamber (ALA Scientific Instruments) in buffer Em D50 (140 mM NaCl, 3 mM KCl, 3 mM CaCl_2_, 1 mM MgCl_2_, 50 mM D-Mannitol, 10 mM HEPES, pH 7.3). Worm heads were illuminated with yellow light (80 mW/cm^2^) using a 590 nm LED and filterset as described above. Calcium traces were taken by a 25× Oil immersion objective (Zeiss LCI Plan-Neofluar 25 × 0.8 Imm Corr DIC) and analyzed by ImageJ, generating ROIs for terminal bulb and background. As a control, worms expressing mCherry in the pharynx (pmyo2::mCherry) were used. Data were calculated to obtain Δ*F*/*F*. Pump events were either scored by eye, judging the slight movements visible in the image stream (for RCaMP), or by analyzing kymographs of line scans through the terminal bulb (mCherry), where the lumen opening could be easily visualized.

### Imaging GECIs in *C. elegans* AWC^on^ sensory neuron

The same experimental protocol was used as in previous GCaMP experiments (Akerboom et al., 2012a). All recordings are from the AWC^on^ sensory neuron. After 10 s of observation, odor (IAA @ 10^−4^ dilution) was added for 5 min. At the last 10 s of odor addition, imaging was commenced for another minute to observe the neuron's response to odor removal (the AWC^on^ neuron activates in response to odor removal).

### Imaging RCaMP1f activity in *Drosophila* motor neuron terminals

#### Genetics and animal preparation

To generate larvae expressing RCaMP in motor neuron terminals, we crossed pJFRC7-7.20XUAS-RCaMP1f flies to a GAL4 driver (OK371-GAL4) that expresses in all glutamatergic neurons (Mahr and Aberle, 2006). Resulting larvae were raised on standard media at 25°C. Wandering third instar larvae were then dissected in physiological saline containing (in mM) 135 NaCl, 5 KCl, 2 CaCl_2_, 4 MgCl_2_, 5 TES, 36 sucrose, pH 7.15. Animals were filleted as described previously (Pulver et al., 2009; Akerboom et al., 2012a) and in each preparation, the central nervous system was dissected away leaving only peripheral nerves and muscles. Larval fillets were then mounted on a standard electrophysiology rig equipped with a gravity fed perfusion system. Saline containing 7 mM glutamic acid was constantly superfused over preparations before imaging to reduce movement artifacts. Temperature was maintained at ~22°C.

#### Stimulation parameters

To stimulate motor axons, cut segmental nerves were drawn into a suction electrode attached to a Model 2100 isolated pulse stimulator (A-M Systems, Sequim, WA). The electrode was maneuvered using a MP-285 micromanipulator (Sutter Instruments, Novato, CA). 2.9 V, 300 μS pulses were delivered at 1, 10, 20, 40, 80, 160 Hz for 2 s durations. Approximately 20 s intervals separated each stimulation frequency; each frequency was repeated at least twice for every terminal examined.

#### Acquisition hardware and software

To visualize RCaMP-expressing motor neuron terminals, we used an Olympus BX51wi compound microscope equipped with a filtered (band bass 572 ± 17.5 nm) white LED light source (OptoLED, Cairn Instruments, Kent, UK) and GFP/mCherry excitation and emission filters (Chroma Technology, Bellows Falls, VT). LED light intensity was kept under 0.37 mW/mm^2^ to reduce bleaching. An Andor DU897 EMCCD camera (Andor Technologies, Belfast, Northern Ireland) was used to capture image data (7.12 fr/s) during nerve stimulations. We simultaneously recorded nerve stimulation times and temperature using a Powerlab 16/30 data acquisition system and Chart 7.1 software (both from AD Instruments, Colorado Springs, Co.). Image data and stimulation times were synchronized off-line using custom scripts in MATLAB (Mathworks, Natick, MA).

#### Image analysis

Motor neuron terminals on muscle 13 (m13) in abdominal segments 3–6 were targeted for analysis. Regions of interest (ROIs) were manually drawn on the 3 most proximal boutons on each m13 muscle. Small lateral shifts in terminal location were corrected off line using image stabilization plugins in ImageJ (Schneider et al., 2012). Percent change in fluorescence from baseline (Δ*F*/*F*) was then calculated for each ROI using custom scripts in MATLAB; intensity values from each ROI were averaged to obtain data for each terminal. A total of eight terminals were measured in four animals (2 terminals per animal). Amplitudes and time courses of ROI signals were analyzed using custom scripts in Spike2 (Cambridge Electronic Design, Cambridge, UK).

### RCaMP activity in zebrafish trigeminal neurons

mitfa^−/−^ (nacre) zebrafish were maintained under standard conditions at 28°C and a 14:10 h light:dark cycle. Larvae were raised in E3 embryo medium (5 mM NaCl, 0.17 mM KCl, 0.33 mM CaCl_2_ and 0.33 mM MgSO_4_). Embryos were injected at the 1 cell stage with 20 ng/μl plasmid DNA encoding RCaMP under the control the pan-neuronal elavl3/HuC promoter (elavl3:RCaMP), and 40 ng/μL Tol2 transposase mRNA diluted in E3 medium with 0.025% Phenol Red. Forty-eight hour post-fertilization embryos showing expression in isolated trigeminal neurons were treated with 1 mg/mL bath-applied alpha-cobratoxin for 15 min to block tail movements. They were mounted in 1.5% low melting temperature agarose, and imaged using an microscope with a 40×/0.8NA objective lens (Olympus) and a CCD camera (Hamamatsu Orca). 1 ms field stimuli were applied using two mesh electrodes in the bath and a stimulator (Grass SD9). Stimulation voltage was calibrated to the threshold to elicit a behavioral response to one pulse in the set up. One spike in a trigeminal neuron has previously been shown to be sufficient to elicit tail movement (Douglass et al., 2008). Software written in LabView (National Instruments) was used for image acquisition and stimulus control. ROI were selected manually, and data was analyzed using MATLAB (MathWorks).

## Author contributions

Jasper Akerboom, Eric R. Schreiter, Loren L. Looger conceived the project. Jasper Akerboom designed and optimized the sensors. Nicole C. Calderón, Karen S. Sarkisyan, Jonathan S. Marvin assisted in library screening. Lin Tian performed the neuron-astrocyte experiments. Sebastian Wabnig, Elisabeth Fischer, Christina Schüler, Alexander Gottschalk performed the integrated optogenetics/functional imaging experiments in worms, as well as the pharyngeal imaging. Matthias Prigge, Peter Hegemann performed the integrated optogenetics/functional imaging experiments in cultured cells. Johan Tolö, Sebastian Kügler performed the mitochondria/cytoplasm imaging. Andrew Gordus, Cornelia I. Bargmann tested RCaMPs in worm chemosensory neurons. Michael B. Orger, Kristen E. Severi analyzed RCaMPs in zebrafish. John J. Macklin, Ronak Patel performed photophysical characterization. Stefan R. Pulver tested RCaMPs in flies. Trevor J. Wardill, Tsai-Wen Chen, Douglas S. Kim, Lin Tian, Nicole C. Calderón, Leon Lagnado performed the electrophysiological calibration of the GECIs in cultured neurons. Jasper Akerboom, Eric R. Schreiter solved the protein crystal structures. Jasper Akerboom, Eric R. Schreiter, Loren L. Looger managed the project.

### Conflict of interest statement

The authors declare that the research was conducted in the absence of any commercial or financial relationships that could be construed as a potential conflict of interest.

## Genbank

RCaMP1h has been deposited in NCBI GenBank (http://www.ncbi.nlm.nih.gov/genbank/) under accession number KC572547.

## Construct availability

RCaMP1h DNA and viruses are available from AddGene (http://www.addgene.org;#70014) or the University of Pennsylvania Vector Core (http://www.med.upenn.edu/gtp/vector_core.shtml), respectively.

## Protein structures

X-ray structures have been deposited in the Protein Data Bank (http://www.rcsb.org). Accession codes are shown in Table 2.

## Abbreviations

AP: action potential
FP: fluorescent protein
GFP: green fluorescent protein
GECI: genetically encoded calcium indicator
FRET: Förster resonance energy transfer
ACh: acetylcholine
ChR2: channelrhodopsin-2
C1V1: ChR1-VChR1 chimera
PAGE: polyacrylamide gel electrophoresis
RMSD: root mean square deviation
SNR: signal-to-noise ratio.
